# Preliminary study on parameterization of raw electrical bioimpedance data with 3 frequencies

**DOI:** 10.1038/s41598-022-13299-7

**Published:** 2022-06-03

**Authors:** C. A. González-Correa, S. A. Jaimes, J. I. Cárdenas-Jiménez

**Affiliations:** 1 Research Group on Electrical Bio-impedance (GruBIE), Universidad de Caldas, Manizales, Colombia; 2 Research Group on Thermal-Dielectric Properties of Composites, TDPC-Group, Universidad Nacional de Colombia, Manizales, Colombia

**Keywords:** Biomedical engineering, Computational science, Software

## Abstract

This study tests the geometrical parameterization method for Electrical Bio-Impedance Spectroscopy (EBIS) readings previously proposed by one of the authors. This method uses the data of just three frequencies (therefore called 3P method). The test was carried out by the analysis of parameterization from 26 spectra (selected from 13 data sets) by the non-linear square (NLS) method, the 3P method and a combination of the two (3P-NLS). Additionally, the behaviour of the 3P method for 4 levels of noise and 3 different ways of segmenting the spectra were also explored with a MATLAB simulation of 400 spectra. Finally, a system for the classification of EBIS readings is presented, based on deviations of the raw data from the semi-circle obtained by the parameterization methods. Overall, the results suggest a very good performance of the 3P method when compared with the other two. The 3P method performs very well with levels of noise of 1 and 2%, but performs poorly with levels of noise of 5% and 10%. The results support the idea that the 3P method could be used with confidence for the parameterization of EBIS spectra, after the selection of three adequate frequencies according to specific applications.

## Introduction

The applications and use of Electrical Bio-impedance Spectroscopy (EBIS) in the biomedical field is growing and there are now multiple areas in human medicine in which it is being used^1,2^. Nevertheless, there are still some issues to resolve, amongst which we would like to mention: accuracy of measurement devices (i.e. different devices may not give the same impedance readings when measuring the same object under identical conditions)^3^, number of frequencies and the best ones to be used^4^, equations selected for calculations^5^ and use of raw data versus parameters^6^.

In respect to the latter issue, our opinion is that parameters should be preferred over raw data^7^. Parameters that fit a model calculated from raw data allow the characterization of the systems being studied^7,8^. In our case, we deal with the Cole model^9^, for which the most common method used for the fitting is nonlinear least squares (NLS), which requires a minimum number of readings equal or greater to the number of parameters to be calculated (which would be four points in this case). Gonzalez-Correa proposed a geometrical approach for fitting EBIS data that, theoretically, would only require the readings at three different frequencies (i.e. three “points”) in the range of the β dispersion^10^. From here on, we will call this approach the 3P (“three points”) method, where the parameters of the dispersion circle are the location of its centre (*x* and *y*) and the length of its radius (*r*). This proposal is mainly based on two facts: (a) the prediction by the Cole model that, for the *β* dispersion, EBIS readings fit to a depressed semicircle^9^ when resistance (the real part, *Z*´ or *R*) and reactance (the imaginary part, *Z*´´ or *X*c) are plotted in the complex plane; and (b) the well-known theorem that “there is only one circle passing through any three given non-collinear points”.

Most EBIS work is carried out in the lower region (5–500 kHz) of the *β* dispersion (1 kHz–10 MHz,), a dispersion mainly attributed to the presence of cell membranes^11^. In this paper, we show the results of testing the 3P approach using data obtained from different sources. For this purpose, we parameterized the data to the Cole model in three different ways: (a) with the 3P method (see Supporting Information 1), (b) with the NLS method using intuitive initial values, and (c) with the NLS method using the data from the 3P method as initial values instead of the intuitive ones (3P-NLS).

Basically, the 3P method proposed by Gonzalez-Correa assumes that: (a) there is a main dispersion in EBIS data better represented for values on the right side of the characteristic frequency (*f*_c_); (b) this region of the dispersion adjusts well to an arch, and (c) the parameters of the circle to which this arch belongs (*x*, *y* and *r*) can be calculated if the coordinate values (*R* and *X*c) of just three points of the arch are known. In this article, it is assumed that, for each reading or spectra, there is just one main dispersion with a circular pattern, which fits well around frequencies in the middle range of the spectrum (i.e. 10–100 kHz). For this dispersion, data points at frequencies 2, 10, and 100 kHz were used with two subsets of data points; frequencies 10, 20 and 50 kHz for another four subsets of data, and data points at frequencies 50, 99 and 495 kHz were used for data taken from^12^, (see Tables S1, Table S2 and Table S3 in Supporting Information 2). The latter authors have proposed a classification of the errors found in spectra of raw data, naming them with the letters A to F, but we propose a new way of classifying raw EBIS data, based on the shape of its plot in the complex plane (presence or absence of the deviations from the arch) and the portions of the arch where they occur. According to^13^, classifications can contribute to the advance of knowledge in science and engineering in, at least, three different ways: “By providing a set of unifying constructs… By understanding interrelationships… By identifying knowledge gaps”. In our case, the origin and significance of the deviations present in many EBIS readings are not well understood and their origins could well be either in the objects being measuring, the equipment or the interface between both. Therefore, a classification provides a common language to name and reference to the different situations found in this field.

We also suggest the possibility of replacing two indices that are being used in EBIS studies, especially in the last decade^14^, namely the phase angle at 50 kHz (*PA*_50_ or *φ*_50_^15^) and the impedance ratio *IR* (ratio between impedance at 200 kHz over impedance at 5 kHz, i.e., *Z*_200_/*Z*_5_), for what we would like to call maximum phase angle (*φ*_max_) or phase angle at the characteristic frequency (*φ*_fc_) and the *IR* for the ratio between impedance at infinite frequency over impedance at zero frequency (*Z*_∞_/*Z*_0_) as used by^15^.

This method could be of interest because it would allow the development of less complex and cheaper instruments optimized for only three frequencies, emulating multi-frequency instruments, just as was suggested by^16^, who developed a method for obtaining the Cole parameters based on 4 points.

Regarding hardware, a system based on three frequencies could be optimized for each of them, using fewer stages of adjusting, compensation and correction than multi-frequency ones. In the latter, this type of optimization is difficult, because of the high selectivity of adjustment circuits such as GIC (General Impedance Converters)^17^. Also, optimizing a multi-frequency device implies a higher cost, a bigger area of printed circuit board and higher power consumption.

Regarding software, the simplicity of the algorithm allows its implementation in low capacity processors such as microcontrollers. Naranjo et al.^18^, implemented an algorithm based on the 3P method in little segments for fitting spectra, developed in a PIC from Microchip.

It is also important to highlight an existing broad interest in the development of low cost, portable and quick response electronic instrumentation for measurement of electric bioimpedance spectroscopy (EBIS) using as few as possible frequencies (inclusive less than 3). As parameterization is of paramount interest, these algorithms ought to allow the calculation of the Cole model. In this order of ideas, it is necessary to mention the research by^19–22^, all of whom have developed low complexity hardware systems using different techniques for measurement and mathematical extraction or optimization of parameters, based in the analysis of time domain signals. These three approaches basically implement techniques based in: tuning of oscillators at two frequencies^19,20^, characterization through a simple triangular signal^21^, and the application of a DC-biased AC signal^22^. However, our research is not focused on the development of new hardware based measuring methods, but in the test the proposal 3P method, for future development of a new software able to parameterize spectra acquired in the traditional way. Therefore, this method could be an alternative to fitting data in MF-BIA (Multi-frequency Impedance Analysis) commercial equipment that has less than 10 frequencies, some of them even using just 3 frequencies^23^.

Finally, it is worth mentioning that there is a recent publication reporting the use of the 3P parameterization method which, adjusting different triplet combinations, looks for the combination that gives the smallest normal error, as the authors define it^24^. This development was published after the publication by^10^ and is based on it.

## Methods

### Data

Data used to test the 3P method in this paper were taken from the following sources: (1) our own archives with impedance spectra obtained with a MF-BIA 4200 Hydra Xitron Analyzer from California-USA, a Tissue Impedance Spectrometer equipment from Sheffield-UK (see Gonzalez-Correa et al.^25^), and a homemade electrical impedance spectroscope, BioZspectra, made in Bucaramanga, Colombia; (2) data provided by the Allers Group, SECA, Colombia (with a SECA mBCA 525, Hamburg, Germany); (3) data provided by the French firm Aminogram (with a Xitron and a BX3), and (4) data taken from^12^. From 13 different sets of readings, we analyzed subsets of 5 readings from each, for a total of 65 spectra and we are showing here the results obtained for the best and worst fitting cases of each subset, giving 26 different spectra, where the best and the worst cases were selected considering the sum of squares results of NLS fitting, using a method and software that will be described in the next section. Except for those readings provided by Aminogram (just five spectra for each set), all other subsets of five readings were randomly selected from the corresponding sets of measurements. For spectra where there were visible deviations from a semicircle, a range of points close to an arch were selected in order to compare the three different fitting approaches used in this paper. In the body of this article, we illustrate the process using a set of our own data, with additional information about it given in Table 1. We call this set of data subset 0 (zero). The corresponding information and results about the remaining 12 sets and subsets are shown in Supporting Information 3. In the Table 1, we specify: (a) number of readings available in the set; (b) total number of frequencies given by the device; (c) number of frequencies used for the parameterization, as those visibly deviating from the arch were discharged; (d) range of frequencies present in the initial spectra; (e) range of frequencies present in the part of the initial spectra used for the parameterization; (f) the three frequencies used for the parameterization with the 3P method; (g) origin of the data; note(s) about the set of data.

**Table 1:** Information about subset 0 of data used in this study.

a) Number of readings (spectra) in the set	20
b) Number of frequencies in the spectra	50
c) Number of frequencies used to compare the fittings	22
**d) Range of frequencies in the spectra**	
Lowest (kHz)	5
Highest (kHz)	1000
**e) Frequencies used in the modelling (EISSA)**	
Lowest (kHz)	5
Highest (kHz)	50
f) Frequencies used for the 3P algorithm (kHz)	10, 20 and 50
g) Source	Own archives from a previous study with a Xitron 4000B
h) Note/s	Whole body measurements on human subjects

### Fitting experimental data

Data from the 65 experimental impedance spectra were parameterized in three different ways, and these three approaches were compared among them, to see if there were statistically significant differences between the parameters and the models obtained with the 3P, the NLS and the 3P-NLS algorithms. NLS fitting was carried out with the free software for impedance data fitting EISSA (EIS Spectrum Analyser) software developed by^26^, using the Levenberg–Marquardt method.

The 3P method consists in the selection (if there are more than 3) or the reading of 3 points of the electrical bioimpedance spectra, the calculation of the three geometrical parameters of the semicircle joining the three points in the complex plane (centre coordinates, *x* and *y*, and length of the radius, *r*), and, from there, the calculation of the Cole parameters (*R*_0_, *R*_∞_, *τ* and *α*), based in the geometrical relations of the model and the semi-circle, which have been reported by^6,26^. The mathematics of the 3P parameterization method and the MATLAB (MATrix LABoratory, by MathWorks version 9.8, R2020a)) code for the calculations are described in Supporting Information 1. Because the modelling of the raw data using the three different methods are very close together, in the graphics we represent different portions of their respective semicircles so that all three can be visualized.

### Quality of the fittings

To evaluate the quality of the fitting, we used two different approaches: the first is the sum of squares of the errors, that we name SS, considering the square of the errors between the modulus of *Z* measured and the modulus of *Z* calculated from the estimated parameters; and the second is the sum of squares radial errors, which we name SSR, considering the square of the difference between the distance from the measured *Z* point to the centre of the circle and the radius of the modelled circle (represented in the complex plane). For further analysis, from each of the 5 readings, we chose the “best” (lowest sum of squares) and the “worst” (higher sum of squares) cases.

### Statistical analysis

Statistical analysis of the results obtained with the three methods mentioned in this article was carried out using the SPSS (Statistical Package for the Social Sciences) package (by IBM v. 26). For this purpose, data were considered in two different ways: (a) all “best” and all “worst” cases, separately, and (b) all data together (“best” and “worst”). Normality was checked with the Shapiro–Wilk test and variance with the Levene’s test. When normality was present, the Friedman test was used to determine if the different sets of data could be considered as equal and, when not, one-way ANOVA (ANalysis Of VAriance) with blocks was applied. When differences between the fitting methods were detected, the Tukey test was further applied.

### Noise analysis of the 3P method

Because the 3P method is not an optimization algorithm, measurement deviations due to instrument inherent errors and the noise will notably affect its performance. Taking this consideration in account, we carried out an assessment to determine its behavior in relation to different noise levels, considering the region of the semi-circumference from where the 3 points for the parameterization were taken. For this purpose, 400 simulated spectra were generated, using MATLAB software, with four levels of random noise uniformly distributed (assuming that the whole frequencies have the same sensitivity against noise): 1%, 2%, 5% and 10% (100 spectra for each level of noise). The parameters of the simulated spectra were selected to obtain one dispersion centred in the beta region (1 kHz–1 MHz). The selected parameters are presented in Tables 6, 7 and 8, together with the results of the noise analysis. For assessment of the effect of the region of the spectra from which the three points are selected, three ways of segmenting the spectra were analysed: (1) one frequency per decade using the entire circumference: 2 kHz, 20 kHz and 200 kHz; (2) the spectra were divided into two halves: 2 kHz, 10 kHz and 30 kHz for the first half, and 30 kHz, 100 kHz and 500 kHz for the second half, and, finally, (3) the spectra were divided into three parts: 2 kHz, 5 kHz and 10 kHz for the first part; 15 kHz, 20 kHz and 50 kHz for middle part, and 200 kHz, 500 kHz and 1 MHz for the last part.

### Sensitivity analysis

In order to perform a sensitivity analysis of the 3P method in relation to coordinate variations, scatter plots were used, using the same spectra generated for the noise analysis and the same ways of point distribution and selection. From there, data fitting was performed based on the parameter trends and this analysis was implemented through a MATLAB developed routine.

### 3P-NLS method efficiency

It is known that the use of good initial parameters to fitting data by NLS methods improves its efficiency, especially in terms of computational cost. To assess the advantages of using 3P parameters as initial values, we carried out the fitting of 26 data with the Levenberg^27^ and Marquardt^28^ method, and the results were compared against intuitive initial values. Again, the fitting was carried out with the EISSA software and the efficiency was measured by the number of iterations.

### Classification of EBIS spectra according to their deviations from an arch

**Figure 1:**
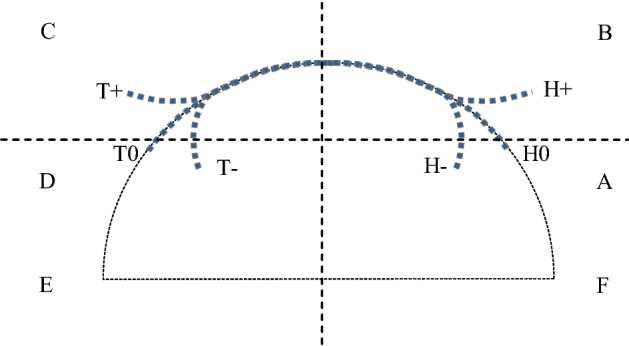
Types of raw data deviations from the semicircle, according to the proposed nomenclature of this study. H stands for head and T stands for tail.

**Figure 2:**
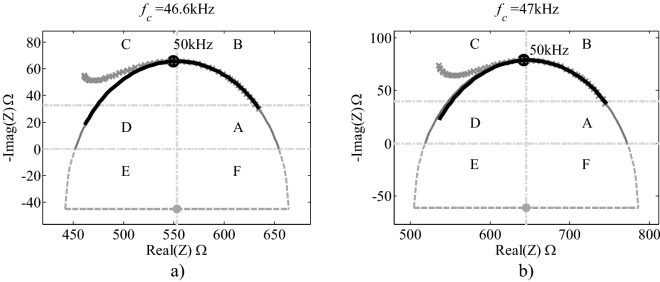
Complex representation (Nyquist plot) of the best (**a**) and the worst (**b**) fittings obtained from subset 0. The three models shown for each spectrum correspond to the data in Table 3. The black, thicker line corresponds to the 3P model (it covers the frequencies of the spectra), the solid thinner line corresponds to NLS method (going from ${R}_{0}$ to ${R}_{\infty }$) and the dashed line corresponds to the 3P-NLS method (it covers the whole semicircle). The shape of both spectra look similar, but their values are not. The modelling with the 3P method gives a curve with a slight inward deviation.

As another contribution of this article, we propose a new way of naming and classifying EBIS spectra, according to the presence or absence of deviations from an arch at lower and higher frequencies (Fig. 1). For this purpose, we divide the right side of the Cartesian two-dimensional (2D) plane, where EBIS spectra are represented, as follows: a vertical line crossing the centre of the circle to which the arch of the EBIS spectra belongs, and a horizontal line intersecting the latter at the midpoint between the *x* axis (*R*) and the impedance value for the characteristic frequency (*f*_c_), i.e., half of the reactance at *f*_c_ or, also, (*r* + *y*)/2. This gives six areas that we would like to name from A to F, as indicated in Figs. 1 and 2.

Each spectrum is then divided into three parts that we would like to designate, from the left (higher frequencies) to the right (lower frequencies) as tail (T), trunk (TH) and head (H). Once the fitting is done, both the head and tail can either fit well to the model or deviate from it. In the latter case, one of them is or both are either inside the circle (deviation towards the centre of the circle or negative deviation) or outside it (deviation away from the circumference or positive deviation). We can, then, classify any specific spectrum according to its positive/negative or no deviations of its head and tail, into nine different categories, as shown in Table 2. Ideal spectra would be those of the type T0/H0 (i.e., with no deviations), which seem to be rather scant in the real world. For more clarity, all four types of deviations are illustrated in Fig. 1.

**Table 2:** Proposed nomenclature for the different types of EBIS spectra.

Tail	+	+	+	0	0	0	−	−	−
Head	+	0	−	+	0	−	+	0	−
Type of curve	T+/H+	T+/H0	T+/H−	T0/H+	T0/H0	T0/H−	T−/H+	T−/H0	T−/H−

Further classification could be achieved adding the location where deviations are present (or where extremes of the spectra end or begin, if there is no deviation). For instance, the two spectra shown in Fig. 2, could be classified as TC+/HA0, indicating that, in both cases, the tail shows a positive deviation (outside the circle) at C, while the head ends in A and lies on the circle (no deviation).

### Proposals for using the 3P method

Adopting the 3P method could have some advantages that we will mention in more detail below. Firstly, it could complement the NLS method or even replace it. Secondly, it would allow an easy estimation of parameters such as what we would like to call maximum phase angle (*φ*_max_ or *PA*_max_), to be used, for instance, instead of the phase angle at 50 kHz commonly used in different studies (see, for instance^29,30^). Thirdly, the impedance ratio *IR* (*Z*_200kHz_/*Z*_5kHz_) used by some authors (see, for instance^15,31^) and incorporated in some equipment like the BiodyXpert by Aminogram, could also be replaced by the ratio (*Z*_∞_/*Z*_0_).

## Results

### Data fitting

Table 3 and Fig. 2 show the best and the worst fittings obtained with the 3 algorithms (3P, NLS and 3P-NLS) for the 5 spectra from a subset of own data used to illustrate the procedure (subset 0). In Table 1 the frequency range used in EISSA software and the selected points for 3P method are shown. In each case, the parameters of the Cole model (*R*_0_, *R*_∞_, *τ*, and α) are given, as well as those of the circle (*x* and *y*, as the coordinates of the centre of the circle, and *r*, as the value of its radius) calculated by each method of fitting. We also divide the plot of the complex plane used in BIA (Nyquist plot) into 6 sub-spaces or locations: A, B, C, D, E and F. As complementary information, *f*c (average of the values obtained with the three methods of fitting) and the location of the point corresponding to 50 kHz are also indicated. In all graphics there are three lines, indicating the three methods employed: a thicker one (3P), a thinner one (NLS) and a dashed one (3P-NLS). In order to differentiate them, the first line is drawn only in the section covered by the raw data, the second goes from *f*_0_ to *f*_∞_, and the third covers the whole semicircle.

**Table 3:** Parameter values for the best and the worst fittings obtained from subset 0. Modelled arches of this example are shown in Fig. 2.

Parameter	3P	NLS	3P-NLS
**Best fitting with the 3P algorithm**			
*x* (Ω)	552.9033	552.9700	552.8850
*y* (Ω)	− 45.5867	− 44.9486	− 45.2311
*r* (Ω)	111.4057	110.6691	110.9531
*R*_0_ (Ω)	654.5551	654.1000	654.2000
*R*_∞_ (Ω)	451.2515	451.8400	451.5700
*τ* (s)	3.4008E − 06	3.4224E − 06	3.4155E − 06
*α*	0.7316	0.7337	0.7327
*f*c (kHz)	46.7991	46.5034	46.5983
**Worst fitting with the 3P algorithm**			
*x* (Ω)	647.8615	645.3000	645.4200
*y* (Ω)	− 58.3636	− 61.7126	− 61.4309
*r* (Ω)	137.2768	140.8673	140.5642
*R*_0_ (Ω)	772.1137	771.9300	771.8500
*R*_∞_ (Ω)	523.6093	518.6700	518.9900
*τ*	3.4642E − 06	3.3463E − 06	3.3541E − 06
*α*	0.7204	0.7113	0.7121
*f*c (kHz)	45.9428	47.5608	47.4504

When the data of all thirteen spectra considered as the best fittings were analysed with the Shapiro–Wilk test, not all the parameters showed a normal distribution (except those for parameter *h*, subsequently examined with an ANOVA), but they all had a homogeneous variance (Levene test). No statistically significant difference between the three methods of fitting was found with ANOVA for *h*, and the Friedman test for the remaining parameters. For spectra considered as the worst fittings, *R*_∞_ and *α* were the sole cases that showed normality but, again, all sets of data had similar variances. When all cases were analysed together (best and worst combined), none of the values for the different parameters had a normal distribution. The analysis of variance in all cases showed that all of them had a homogeneous variance.

Table 4 shows the *p*-values for the comparison of the three fitting methods used in this study for each of the fitting parameters considered. Only *R*_∞_, when the worst cases were considered, and *x*, when all cases (worst and best) were analyzed together, showed a statistically significant difference. In both cases, the Tukey test indicated that average values obtained by the NLS method were lower than the average values obtained with the 3P method, although they were equal to the values obtained with the 3P-NLS. In turn, the values obtained with the 3P method were statistically equal to those obtained with the 3P-NLS method.

**Table 4:** *P*-values for the comparison of the parameters obtained for the best, the worst and both cases considered together with the spectra taken from all subsets of data (0–12).

Parameter	Best	Worst	Best + worst
*x* (Ω)	0.094569	0.31757	**0.01346**
*y* (Ω)	0.825728	0.08374	0.34064
*r* (Ω)	0.839457	0.13236	0.24097
*R*_∞_ (Ω)	0.599179	**0.02428**	0.08128
*R*_0_ (Ω)	0.43888	0.36788	0.91633
*α*	0.807118	0.06750	0.60653
*τ* (s)	0.517071	0.85594	0.22313
*f*_c_ (kHz)	0.395118	0.67312	0.22313

Significant values are in bold.

### Quality of the fittings

Table 5 shows the average percentage of the residuals given after the modelling performed using the three different methods, with the two different ways of calculating them, as previously explained (SSR and SS). In Supporting Information 4 we show how these percentages are calculated.

**Table 5:** Maximum error, average and standard deviation (SD) of the residuals obtained for the two methods of calculating them (SSR and SS) after the fitting of the data with the three different methods of fitting (3P, NLS and 3P-NLS).

	Best			Worst		
	3P (%)	NLS (%)	NLS-3P (%)	3P (%)	NLS (%)	NLS-3P (%)
Max %SSR	1.00	1.42	1.15	2.30	1.69	1.69
Average %SSR	0.55	0.51	0.46	0.98	0.84	0.87
SD %SSR	0.23	0.34	0.28	0.58	0.46	0.48
Max %SS	2.34	1.62	1.62	4.55	3.94	3.94
Average %SS	0.45	0.32	0.32	0.98	0.73	0.74
SD %SS	0.68	0.51	0.51	1.56	1.28	1.27
Max % SSR & %SS	2.34	1.62	1.62	4.55	3.94	3.94
Average % SSR & %SS	0.50	0.41	0.39	0.98	0.78	0.81
SD % SSR & %SS	0.50	0.44	0.41	1.15	0.94	0.94
%Average% + %SD SSR	0.77	0.85	0.74	1.57	1.30	1.35
%Average% + %SD SS	1.13	0.83	0.83	2.54	2.01	2.02
%Average% + %SD SSR & SS	1.00	0.85	0.80	2.13	1.73	1.75

In the Table 5, the maximum error as well as the average and standard deviation (SD) for each method are given in three different ways: SSR and SS separated and, then, considering them all together. The lowest three rows show the sum of the average plus the SD to give an idea of how big the majority of the errors were.

### Noise performance

**Figure 3:**
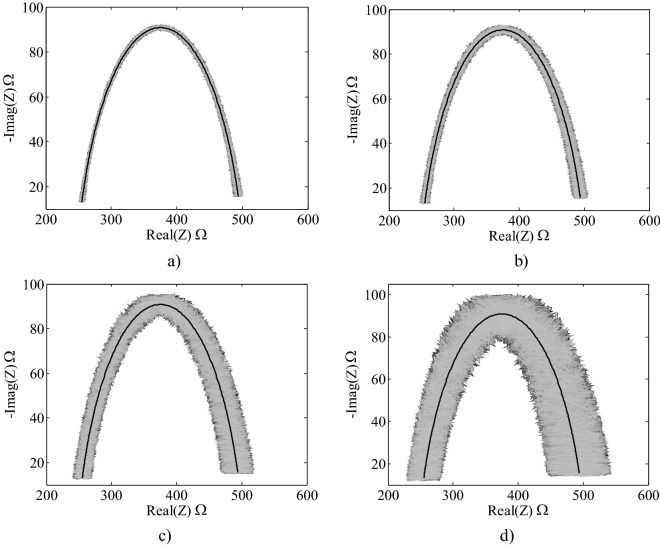
Complex representation of the noisy spectra at different noise levels, (**a**) 1%, (**b**) 2%, (**c**) 5%, (**d**) 10%. The middle solid lines in black represent the spectrum without noise.

All noisy spectra generated for the analysis are presented In Fig. 3, in a superposition that allows a visual perception of the ranges in which the points on the complex plane fluctuate.

Tables 6, 7 and 8 show the values obtained for the four Cole parameters using the 3P algorithm with simulated data with four different levels of noise (1, 2, 5 and 10%) and with three different forms of selecting the portions of the spectra.

**Table 6:** Parameters obtained by the 3P method with simulated spectra created with different percentages of added noise. Form 1.

Parameter	Noise level (%)				Theoretical value
	1	2	5	10	
Mean *R*_0_	500.86	499.84	500.72	497.75	500
Mean *R*_inf_	249.77	250.02	251.19	255.78	250
Mean *τ*	5.66E − 06	5.63E − 06	5.71E − 06	5.80E − 06	5.60E − 06
Mean *α*	0.7977	0.8016	0.8115	0.8005	0.8
SD *R*_0_	3.54	6.93	17.05	36.55	
SD *R*_inf_	2.26	4.67	11.49	22.53	
SD *τ*	1.90E − 07	3.93E − 07	1.00E − 06	2.24E − 06	
SD *α*	1.47E − 02	2.96E − 02	8.29E − 02	1.13E − 01

**Table 7:** Parameters obtained by the 3P method with simulated spectra created with different percentages of added noise. Form 2.

Parameter	Noise level (%)				Theoretical value
	1%	2%	5%	10%	
**First half (lower frequencies)**					
Mean *R*_0_	501.26	500.17	501.31	513.23	500
Mean *R*_inf_	246.19	244.81	225.52	− 10,391.49	250
Mean *τ*	5.55E − 06	5.50E − 06	4.38E − 06	− 7.25E − 05	5.60E − 06
Mean *α*	0.7903	0.7968	0.7364	0.4956	0.8
SD *R*_0_	4.83	9.22	23.19	114.44	
SD *R*_inf_	15.52	28.04	106.68	99,894.24	
SD *τ*	4.42E − 07	7.71E − 07	2.72E − 06	5.12E − 04	
SD *α*	4.84E − 02	8.57E − 02	1.45E − 01	3.67E − 01	
**Second half (higher frequencies)**					
Mean *R*_0_	500.42	505.22	525.20	621.43	500
Mean *R*_inf_	249.98	249.71	249.06	129.50	250
Mean *τ*	5.71E − 06	6.15E − 06	9.88E − 06	3.77E − 02	5.60E − 06
Mean *α*	0.8006	0.7959	0.7671	0.6475	0.8
SD *R*_0_	13.27	26.22	78.43	807.69	
SD *R*_inf_	2.60	4.77	13.20	478.23	
SD *τ*	8.47E − 07	1.88E − 06	9.37E − 06	3.72E − 01	
SD *α*	3.51E − 02	6.38E − 02	1.29E − 01	2.45E − 01

**Table 8:** Parameters obtained by the 3P method with simulated spectra created with different percentages of added noise. Form 3.

Parameter	Noise level (%)				Theoretical value
	1	2	5	10	
**First third (lower frequencies)**					
Mean *R*_0_	697.64	1930.62	548.61	512.44	500
Mean *R*_inf_	− 1352.06	− 132.88	381.61	421.85	250
Mean *τ*	− 4.28E − 03	− 1.71E + 00	3.66E − 05	5.71E − 06	5.60E − 06
Mean *α*	0.7493	0.6529	0.3222	0.2197	0.8
SD *R*_0_	952.46	13,727.78	217.06	178.74	
SD *R*_inf_	14,063.78	3371.96	226.46	151.54	
SD *τ*	3.11E − 02	1.71E + 01	6.39E − 04	8.66E − 05	
SD *α*	1.58E − 01	2.74E − 01	4.80E − 01	4.89E − 01	
**Middle third**					
Mean *R*_0_	498.93	502.22	487.87	484.40	500
Mean *R*_inf_	250.92	247.80	258.54	255.97	250
Mean *τ*	5.61E − 06	5.39E − 06	3.13E − 06	1.12E − 06	5.60E − 06
Mean *α*	0.8106	0.7590	0.4895	0.3544	0.8
SD *R*_0_	17.83	35.37	144.25	205.31	
SD *R*_inf_	16.22	33.18	149.30	204.56	
SD *τ*	2.30E − 07	1.62E − 06	5.88E − 06	1.70E − 05	
SD *α*	8.41E − 02	1.39E − 01	4.70E − 01	5.03E − 01	
**Last third (higher frequencies)**					
Mean *R*_0_	476.75	353.17	289.55	752.48	500
Mean *R*_inf_	91.96	− 322.14	177.02	− 75.70	250
Mean *τ*	2.47E − 05	7.27E − 06	1.08E − 06	1.18E + 03	5.60E − 06
Mean *α*	0.6909	0.4680	0.2706	0.0888	0.8
SD *R*_0_	938.60	302.39	100.11	4751.56	
SD *R*_inf_	428.00	4883.04	501.85	2999.99	
SD *τ*	1.85E − 04	4.48E − 05	9.14E − 06	1.18E + 04	
SD *α*	2.36E − 01	4.02E − 01	4.75E − 01	3.44E − 01	

### Sensitivity analysis

**Figure 4:**
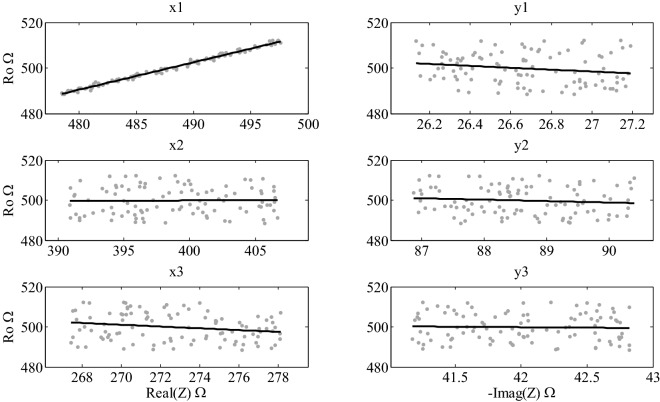
Scatter plots for parameter ${R}_{0}$, at 2% of noise, Form 1.

In Fig. 4, scatter plots for the parameter *R*_0_ with a noise level of 2% is presented for form 1 of point distribution. The figures for the parameters ${R}_{\infty }$, $\alpha$ y $\tau$ in this same configuration are presented in Supporting Information 6. Additionally, results for the 6 different ways of selecting the points and the four noise levels considered in this study are also presented in the tables of the same support information.

### Fitting efficiency

Table 9 shows the mean number of iteration need for intuitive initial parameters and the parameters of the 3P method.

**Table 9:** Mean number of iterations needed for the NLS fitting using initial intuitive values versus fitting using the values provided by the 3P method as initial values.

	Mean	SD
NLS (intuitive values)	333.5	731.6
3P-NLS	256.8	544.7
Reduction (%)	23.0	25.6

### Spectra classification

If the proposed classification is to be used, the exact points that deviate from the model would have to be mathematically calculated, after establishing a tolerance percentage of deviation (for instance, 1 or 2%). Nevertheless, we would like to point out that, as shown in Supporting Information 2, most of the curves analysed have a positive C tail (TC+), i.e. the left part of the spectra deviates outwards from the circle, while most of them have a zero A head (H0). Only one spectra (best fitting of subset 1) seems to have an ideal behaviour, that is, the whole spectra adjusts well to the calculated model. As an example, already mentioned, both curves of subset 0 seem to be TC+/HA0 curves (see Fig. 2). Some spectra may be difficult to classify, when one of the extremes (either the head or the tail, or both), for instance, first goes outside and then moves towards the circle or vice versa.

## Discussion

In the cases that we are using to illustrate the procedure of the study, it can be seen that the three methods give very similar results for the two cases considered (best and worst fitting), even in the latter. It is also clear that raw data in sub quadrant B adjust very well to the arch, while there are clearly deviations from it, predominantly in the left part of the spectra (higher frequencies). This deviation of experimental data from a fitting arch at higher frequencies, has been considered as an artefact due to the measurement devices and is usually called the “hook effect”^12,32–34^. When comparing the parameter values obtained with three methods, in most cases they can be considered as equal, except for two cases, as shown in Table 4. These results give us confidence that the 3P method is robust and could eventually replace the NLS method for the parameter calculation of the Cole model from raw bioimpedance data. But, even if that were not the case, the fact that there are no statistically significant differences among the NLS and the 3P-NLS methods of parameterization, implies that the 3P method could, at least, provide the initial values for the NLS, which could facilitate the use of the latter, as these values can be obtained automatically, without the need of direct human intervention.

Regarding the quality of the three methods used in this work, it can be seen (Table 4) that the average percentages of the residuals are all below 1%, a value that can be considered as completely acceptable. The average percentages of the residuals and the corresponding standard deviations calculated with the SS method are larger than those obtained with the SSR method due to the fact that the reference values (the 100% values) are much larger for the former (*Z*) than for the latter (*r*).

In relation to noise performance, Table 6 shows that the 3P method performs very well (mean values of the parameters are very close to the theoretical values) when one point per decade is selected for all noise levels, including 5% and 10%, which can be considered as very high and very unlikely in actual measurements. In Fig. 3, it can also be seen that, for levels of noise of 5% and 10%, the variability in the measurement is very high in comparison to the actual spectra given by the instruments that were used for the measurements used in this study.

When parameters are obtained with frequencies in the lower and higher portions of the frequency spectra, the results seem to perform better when lower frequencies are used (Table 7). For 1% and 2% levels of noise the mean values obtained are very close to the theoretical values. When the spectra are divided in three portions, first (lower frequencies), second (middle frequencies) and third (higher frequencies), only those obtained with the middle frequencies are acceptable (Table 8), where the results are good for 1% and 2% levels of noise, relatively acceptable for the 5% level, and very poor for the 10% level.

It is worth highlighting that the results shown in Tables 6, 7 and 8 included all data, even those that did not adjust to the model. Therefore, if some restrictions were considered, in order to reject those spectra not giving valid results, the parameters would have a still better behaviour. Some of the restrictions could be:

- If the three points taking for the modelling at frequencies $f1, f2 y f3$ are defined as $P1=R1+jX1 \; at \; f1; \quad P2=R2+jX2 \; at \; f2 \; and \; P3=R3+jX3 \; at \; f3;$ where $f1>f2>f3$, any spectrum is potentially valid only if $R1>R2>R3$.
- $P1, P2\;\mathrm{and}\;P3$ can not be collinear.
- The coordinates of the center of the semicircle passing through $P1, P2 y P3$ has to be in the fourth quadrant, i.e., $h>0 y k<0$.
- $${R}_{0}>{R}_{\infty }$$
- $${R}_{\infty }>0$$
- Additionally, another selection criterion could be established based on statistical parameters.

The results of the calculations of the parameters using noisy data are shown in Supporting Information 5 (as they were presented in Tables 6, 7 and 8), but, this time, taking in consideration the above mentioned restrictions. Where some data are discarded, the statistical criterion for the elimination of the parameters are those outside quartiles Q1 and Q3. It can be seen, then that, for Form 1, with 1% and 2% noises, no data are discarded and, therefore, the results are equal to those presented in Table 6, indicating a good behavior of all parameters and showing low sensitivity to noise. For noise level of 5%, there are 51 spectra discarded and 60 for noise level of 10%. It can be seen, there, that there is a greater deviation of the mean, but les variability when compared to the initial analysis without data being discarded (see Table S17).

In Form 2, at noise level of 1%, there are no spectra discarded and for noise level of 2%, there are just 2 spectra rejected for the first half, and, then, it can be considered that, at these levels of noise, there are no significant differences when the results are compared to those given by the analysis without discarding data. For noise levels of 5% and 10%, there are data discarded in both halves and, according to the results presented in Table S18, there are significant improvements in the results, both in the means and in the standard deviations.

In form 3, there are spectra discarded in all configurations, something that was expected, given the fact that the narrower the range of distribution of the points, the greater is the sensitivity of the parameters in relation to the level of noise.

Table S19 shows how, using the above mentioned restrictions, the results improve notably for the three configurations a well as for all noise levels. The middle section continues to show the best behavior, especially with noise levels of 1% and 2%. On the sides, data on the right show a better performance of the modelling, for all parameters, at level of noise of 1%, while on the left side the deviations are much greater and would not be recommended for any level of noise.

Based on noise analysis, with and without discards, it can be seen that the greater the distance between the selected points, the better behavior the modelling shows, for all levels of noise. Parameters ${R}_{0}$ y ${R}_{\infty }$ are the ones less sensitive to noise for the majority of configurations, with exception of the sides in form 3. In this case, it can be noticed that for the right side, the parameter less sensitive to noise is ${R}_{0}$, while, for the left side, the parameter less sensitive to noise is ${R}_{\infty }$. This was expected, given the proximity of the points to the respective geometrical area of the semicircle. The parameter $\alpha$ shows a good behaviour for all configurations at levels of noise of 1% and 2%. For levels of noise of 5% and 10%, it shows a good behavior only in Form 1. Finally, the parameter $\tau$ is the most sensitive to noise, due to its dependence of the coordinates of the three selected points taken for the modelling, their frequencies and the other three parameters of the Cole model. However, it has a good behavior in Form 1 at all noise levels, as well in Form 2 and Form 3 at levels of noise of 1% and 2%.

Using scatter plots, it can be seen that there are linear trends between the parameters of the Cole model and the coordinates of the impedance points. In Fig. 4, it can be seen that there is an almost perfect linear correlation between ${R}_{0}$ and abscise × 1. The fitting data gave a relation of ${R}_{0}=1.005x1-85.76$, with a Pearson correlation $r=0.99485$. Additionally, it can also be observed that there are some other slightly tendencies between abscise of X3 and the ordinate y1. For the rest of the coordinates, trends are almost imperceptible. Based on the figures of Section VI, in “Supporting Material”, we can say that ${R}_{\infty }$ presents a high correlation with abscise × 3 (${R}_{\infty }=1.2763x3-98.077, r=0.89938$), while $\alpha$ y $\tau$ are highly correlate with × 1 ($\alpha =-0.0044968x1+2.9951, r=-0.87196$; $\tau =\left(5.90E-8\right)x1-2.32E-5, r=0.86268$). Additionally, results on Table S20 shows that, for the different levels of noise considered in this study, the trends are the same as those already mentioned. In this way, for this configuration, the Cole parameters are predominantly sensitive to × 1 y × 3.

Results for all configurations and all levels of noise are shown in Tables S20 to S25. For Form 2 on the right side, ${R}_{0}$ is highly correlated to × 1; ${R}_{\infty }$, $\alpha$ and $\tau$ are highly correlated to × 1 and × 2. Fort he left half, ${R}_{0}$ shows a high correlation to × 2, ${R}_{\infty }$ to × 3, and $\alpha$ and $\tau$ to × 2 and × 3.

There are no noticeable correlations of the parameters to the coordinates in Form 3. In this case, there are moderate relations of each parameters to the abscises and the ordinates. This can be explained by the fact that division in thirds is the most susceptible to variation in the location of the 3 points. However, similar to the noise level, the middle or center zone is the one that shows higher levels of correlation to a single coordinate.

When using the values obtained with the 3P methods as initial values for the parameterization with the SS method, Table 8 shows that it needs 23.0% fewer iterations with a 25.6% narrower SD. This, then, indicates that, given the fact that both the NLS and the 3P-NLS methods give equal results, the use of the 3P could signify a quicker method, on top of allowing an automatic calculation of the initial values for the NLS method. Additionally, in order to define the parameters to be used as initial values, the restrictions considered above can be used. In the case that data are given as a series of measurements o repetitions, the standard deviation of the parameters could be used as a validation criterion. Finally, the spectra classification proposed in this article could be more useful than the one proposed by^12^, as it allows a better understanding of their behaviour.

## Conclusions

This work was meant to test the proposal of a geometrical method for the parameterization of bioimpedance measurements by Gonzalez-Correa^10^, using data from just three frequencies (3P method). The results suggest a very good performance of the method with the advantage of being easily implemented. If the NLS is preferred, the 3P method could improve the process, not only providing initial values that can be automatically calculated by software, without direct human intervention, but also reducing the average number of iterations needed, and giving narrower standard deviatons. There is also potential to improve the 3P method by taking a couple of repeated measurements and averaging them, in order to minimize the impact of noise. Probably 3 to 6 repeated readings could do the work.

The results presented here suggest the following considerations if the 3P is to be used: (a) the wider the 3 points selected are separated the better the fitting; (b) the first and the last thirds of the spectra are not recommended, as small deviations of the data would produce very different circumferences; (c) data from the middle third show a good behaviour to the noise.

The more adequate frequencies to be used for specific applications should be selected according to the response of the specific objects to be measured. Once the parameters of the circle for each spectrum are obtained, the calculation of other variables as *R*_0_, *R*_∞_, *φ*_max_ and *f*_c_ can be very easily done. These variables could prove to be useful in future studies.

In order to overcome the possible inconvenience of the potential high sensitivity of using just 3 points for the parameterization process, instead of measuring several frequencies, taking three or even more consecutive measurements at just three pre-established frequencies, and, then, averaging them, could be an appropriate approach. Another possibility would be to take more than three points (frequencies), calculating the parameters with all possible combinations of 3 points and averaging them (for 4 points or frequencies there would be 4 combinations, for 5 points 10 combinations, and for 6 points 20 combinations).

Finally, the sensitivity analysis by means of scatter plots could also be a very useful tool when the 3P method is to be used, both for adequate selection of the points, as wells as for hardware development for specific application in the field of electrical bioimpedance.

## Supplementary Information

Supplementary Information.

## Data Availability

The datasets used and/or analyzed during the current study are available from the corresponding author on request, except for the Aminogram datasets (these data are, however, available from the authors upon request and with permission of Aminogram).
