# Supplementary material for: Preliminary study on parameterization of raw electrical bioimpedance data with 3 frequencies
Source: Sci Rep. 2022 Jun 3;12:9292. doi: 10.1038/s41598-022-13299-7 (PMC9166711; doi:10.1038/s41598-022-13299-7)
Supplement: Supplementary file 1 — Supplementary Information. [file 41598_2022_13299_MOESM1_ESM.pdf]

# Preliminary study on parameterization of raw electrical bioimpedance data with 3 frequencies

C.A. González-Correa[1], *S.A. Jaimes[2], and J.I. Cárdenas-Jiménez[3]

[1]Research group on Electrical Bio-Impedance (GruBIE), Universidad de Caldas, Colombia, South America
[2]Research group on Electrical Bio-Impedance (GruBIE), Universidad de Caldas, Colombia, South America
[3]Research group on Thermal-Dielectric Properties of Composites, TDPC-Group, Universidad Nacional de Colombia, Manizales Campus, Colombia, South America

*CORRESPONDENCE E-MAIL: SAMUELJAIMES2005270@YAHOO.COM

## I. SUPPORTING INFORMATION 1.

### A. *Mathematical description*

The Cole equation is presented as equation (1), which allows the modelling of the electrical response of a tissue presenting just one dispersion.

$Z = R_\infty + \frac{R_0 - R_\infty}{1+(j\omega\tau)^\alpha}$ (1).

Where:

$Z$ is the impedance measured from the biological tissue;
$\omega$ is the angular frequency;
$R_\infty$ is the resistance of the tissue at infinity frequency (resistance of the whole volume, i.e., extra- and intra-cellular spaces);
$R_0$ is the resistance of the tissue at zero frequency (resistance of the extracellular space);
$\tau$ is the time constant or relaxation time of the tissue, a variable dependant of the capacitive effect of the cell membranes and the resistances of both the intra- and the extra-cellular spaces;
$\alpha$ is a measure of the relaxation time distribution, which can be related to different phenomena as, for instance, cellular or molecular interactions, cell size, anisotropy or heterogeneity of the tissue, among others [1]–[3].

This equation corresponds to a semi circumference in the complex plane, and, therefore, a relation between its parameters and those of the semi circumference can be obtained, as it has been reported by [4] and [5].

The three parameters of the circle are named, here, as $h$ (abscise), $k$ (coordinate) and $r$ (radius).

The method used for the parameter estimation is as follows:

Three points of the impedance spectra are taken in the complex plane and the circle

equation calculated.

$R_0$ and $R_\infty$ are calculated using the parameters of the circumference (centre and radius, i.e., $h$, $k$ and $r$), while α is calculated using the parameters $h$ and $r$).

τ is calculated replacing $R_0$, $R_\infty$ and $\alpha$ in (1) and using the selected impedance values and their respective frequencies.

For the calculation of the equation of the circle passing through the three selected points, the starting point is given for the general equation of the circle (equation 2), with three unknown values (h, k and r). As there are three known points [($x_1$, $y_1$), ($x_2$, $y_2$) and ($x_3$, $y_3$)], a three equation system with three unknowns can be formulated:

$$(x-h)^2+(y-k)^2=r^2 \text{ (2).}$$

Equation (2) can be rewritten as:

$$x^2+y^2+Ax+By+C=0 \text{ (3).}$$

Where

$$A=-2h; B=-2k \; y \; C=h^2+k^2-r^2 \text{ (4).}$$

Replacing the three points in equation (3), it gives:

$$\begin{cases} Ax_1+By_1+C=-(x_1^2+y_1^2) \\ Ax_2+By_2+C=-(x_2^2+y_2^2) \\ Ax_3+By_3+C=-(x_3^2+y_3^2) \end{cases} \text{ (5).}$$

Solving system (5) and replacing the corresponding values in equation (4), the parameters of the circle ($h$, $k$ and $r$) are obtained, and, by the Pythagorean Theorem, $R_0$ and $R_\infty$ are calculated as:

$$R_0=h+\sqrt{r^2-k^2} \text{ (6), and}$$

$$R_\infty=h-\sqrt{r^2-k^2} \text{ (7).}$$

The parameter $\alpha$ equals to:

$$\alpha=1\pm\emptyset \text{ (8),}$$

where $\emptyset$ is the normalized angle formed by the abscise and the radius touching $R_\infty$. In other words:

$$\alpha=1\pm\frac{2}{\pi}\operatorname{atan}\left(\frac{k}{\sqrt{r^2-k^2}}\right) \text{ (9).}$$

Expressions (6) to (9) were calculated and adapted from the definitions reported in [4] and [5].

Parameter $\tau$ is not geometry dependant and, therefore, the impedance values are used in order to find it. Rearranging equation (1), it follows that:

$$\tau = \frac{1}{j\omega}\left(\frac{R_0 - R_\infty}{Z - R_\infty} - 1\right)^{1/\alpha} \quad (10).$$

Here, replacing $Z$, its corresponding frequency ($\omega$) and the previously obtained Cole parameters, the value for $\tau$ is calculated. Due to possible measurement errors and the trunction process in the handling of the data, the result is complex and therefore, only the real part is taken into account. Equation (10) can be easily evaluated by means of either a computer or a processor. Nevertheless, in order to facilitate the calculation without the use of complex numbers, following expression applies to the real part:

$$\Re(\tau) = \frac{1}{\omega}\left(\sqrt{\frac{(R_0 - R_Z)^2 + I_Z{}^2}{(R_Z - R_\infty)^2 + I_Z{}^2}}\right)^{1/\alpha} \cos\left[\frac{1}{\alpha}\left\{\operatorname{atan}\left(-\frac{I_Z}{R_0 - R_Z}\right) - \operatorname{atan}\left(\frac{I_Z}{R_Z - R_\infty}\right)\right\} - \frac{\pi}{2}\right] \quad (11),$$

Where $R_Z$ and $I_Z$ correspond to the real and the imaginary parts of the impedance measured, respectively, as indicated in equation (12).

$$Z = R_Z + jI_Z \quad (12).$$

Given that there are three points or impedance measurements, a value for $\tau$ for each one is found and then the three values are averaged.

Equations (10) and (11) were obtained from complex algebraic deductions from the Cole equation.

A continuación se presenta el código desarrollado para realizar los cálculos el cual fue implementado en MATLAB.

```
clear M;
clear Z;
clear i;
clear ZD;
clear ZD1;
clc


%Matriz with one Spectra, ZD1(:,1) REAL; ZD1(:,2) -IMAG; ZD1(:,3) FREQUENCY
ZD1=[310.28  2.0   1000
309.8   2.9   2000
308.23 5.2   5000
305.85 7.9   10000
301.68 11.1 20000
292.93 14.4 50000
284.9   14.8 100000
276.95 13.0 200000
267.28 7.6   500000];
```

```
P1=4;%Number of selected points for 3P calculations
P2=5;
P3=6;

ZD(:,1)=ZD1(:,3);%Matrix with data reordered
ZD(:,2)=ZD1(:,1);
ZD(:,3)=-ZD1(:,2);
ZD(:,4)=abs(ZD(:,2)+i*ZD(:,3));
ZD(:,5)=atan(ZD(:,3)./ZD(:,2))*180/pi;

ZE(1)=ZD(P1,2)+i*ZD(P1,3);%Impedance at selected points
ZE(2)=ZD(P2,2)+i*ZD(P2,3);
ZE(3)=ZD(P3,2)+i*ZD(P3,3);

fE(1)=ZD(P1,1);%frequencies at selected points
fE(2)=ZD(P2,1);
fE(3)=ZD(P3,1);


%%%%%%%%%%%%%%%%%%%%%%%%%%%Calculations of the semicircle parameters h,k and r
    x1=real(ZE(1));
    y1=imag(ZE(1));

    x2=real(ZE(2));
    y2=imag(ZE(2));

    x3=real(ZE(3));
    y3=imag(ZE(3));

    A=[x1 y1 1;x2 y2 1;x3 y3 1];
    B=-[(x1^2+y1^2) y1 1;(x2^2+y2^2) y2 1;(x3^2+y3^2) y3 1];
    C=[(x1^2+y1^2) x1 1;(x2^2+y2^2) x2 1;(x3^2+y3^2) x3 1];
    D=-[(x1^2+y1^2) x1 y1;(x2^2+y2^2) x2 y2;(x3^2+y3^2) x3 y3];

    A1=det(A);
    B1=det(B);
    C1=det(C);
    D1=det(D);

    cx=-B1/(2*A1);
    cy=-C1/(2*A1);

    r=sqrt((B1^2+C1^2-4*A1*D1)/(4*A1^2));

    X=cx-r:0.1:cx+r+0.1;
    M=A1*(X.^2)+B1*X+D1;

    Y1=(-C1+sqrt(C1^2-4*A1*M))/(2*A1);


% ////////////////////Calculations of Cole-Cole
%/////////////////////parameters
h=cx
```

```
k=-cy
radio=r

Rinf=h-sqrt(radio^2-k^2)%R at infinity frequency
Ro=h+sqrt(radio^2-k^2)%R at zero frequency

alpha_exP=1+(2/pi)*atan(1/sqrt((radio/k)^2-1));% alpha calculations
alpha_exN=1-(2/pi)*atan(1/sqrt((radio/k)^2-1));

if alpha_exP<1
  alpha_ex=alpha_exP
else
  alpha_ex=alpha_exN
end


for k=1:3%tao calculations

    A=real(ZE(k));
    B=imag(ZE(k));

    tao_i=(1/(i*fE(k)*2*pi))*((Ro-Rinf)/(A+i*B-Rinf)-1)^(1/alfa_ex);

    tao_v(k)=tao_i;

end

tao=real(sum(tao_v)/3)%tao


%Z calculation from parameter obtained
f=logspace(3,6,100);
w=2*pi*f;
S=i*w;
Zcalc=Rinf+(Ro-Rinf)./(1+(S*tao).^alpha_ex);


%Graphical
subplot(311)%Real part vs frequency
semilogx(ZD1(:,3),ZD1(:,1),'.','color',[0,0,0],'MarkerSize',15)
hold on
semilogx(f,real(Zcalc))
subplot(312)%-imaginary part vs frequency
semilogx(ZD1(:,3),ZD1(:,2),'.','color',[0,0,0],'MarkerSize',15)
hold on
semilogx(f,-imag(Zcalc))
subplot(313)%Complex plane (Nyquist)
semilogx(ZD1(:,1),ZD1(:,2),'.','color',[0,0,0],'MarkerSize',15)
hold on
semilogx(real(Zcalc),-imag(Zcalc))
```

# II. SUPPORTING INFORMATION 2.

TABLE S1.
SOURCES OF THE DATA SETS USED IN THE STUDY

| | Source | Set size | Notes |
|---|---|---|---|
| 1 | Own archives | 60 | Mark 3 (Sheffield-UK), measurements on different vegetables. |
| 2 | Own archives | 42 | BioZpectra (Bucaramanga-Colombia), measurements on different vegetables. |
| 3 | Own archives | 200 | mBCA 525, whole body measurements on humans. |
| 4 | Aminogram | 5 | BX3 subjects sitting, whole body measurements on humans. |
| 5 | Aminogram | 5 | BX3 subjects lying, whole body measurements on humans. |
| 6 | Aminogram | 5 | Xitron subjects lying, whole body measurements on humans. |
| 7 | Ayllón *et al* 2006 | 340 | Error type A. |
| 8 | Ayllón *et al* 2006 | 220 | Error type B. |
| 9 | Ayllón *et al* 2006 | 50 | Error type C. |
| 10 | Ayllón *et al* 2006 | 400 | Error type D. |
| 11 | Ayllón *et al* 2006 | 140 | Error type E. |
| 12 | Ayllón *et al* 2006 | 30 | Error type F. |

TABLE S2.
NUMBER AND RANGE OF THE FREQUENCIES FROM EACH SET OF MEASUREMENTS USED IN THE STUDY.

| | Number of | Range of frequencies in the spectra | |
|---|---|---|---|
| Set | Frequencies | Lowest (kHz) | Highest (MHz) |
| 1 | 8 | 1 | 0.2 |
| 2 | 8 | 1 | 0.2 |
| 3 | 9 | 1 | 0.5 |
| 4 | 54 | 1 | 1 |
| 5 | 54 | 1 | 1 |
| 6 | 50 | 5 | 1 |
| 7 | 256 | 19.4 | 6.3 |
| 8 | 256 | 19.4 | 6.3 |
| 9 | 256 | 19.4 | 6.3 |
| 10 | 256 | 19.4 | 6.3 |
| 11 | 256 | 19.4 | 6.3 |
| 12 | 256 | 19.4 | 6.3 |

TABLE S3.
INFORMATION FROM THE SUBSETS TAKEN FOR THE STUDY.

| Set # | Number of frequencies used to compare the fittings | Range of frequencies used in the modelling | | Frequencies used for the 3P algorithm (kHz) |
|---|---|---|---|---|
| | | Lowest (kHz) | Highest (kHz) | |
| 1 | 6 | 2 | 100 | 2, 10 & 100 |
| 2 | 6 | 2 | 100 | 2, 10 & 100 |
| 3 | 6 | 2 | 100 | 10, 20 & 50 |
| 4 | 22 | 5 | 50 | 10, 20 & 50 |
| 5 | 22 | 5 | 50 | 10, 20 & 50 |
| 6 | 22 | 5 | 50 | 10, 20 & 50 |
| 7 | 144 | 19.4 | 495.0 | 50.0, 98.9 & 495.0 |
| 8 | 144 | 19.4 | 495.0 | 50.0, 98.9 & 495.0 |
| 9 | 144 | 19.4 | 495.0 | 50.0, 98.9 & 495.0 |
| 10 | 144 | 19.4 | 495.0 | 50.0, 98.9 & 495.0 |
| 11 | 144 | 19.4 | 495.0 | 50.0, 98.9 & 495.0 |
| 12 | 144 | 19.4 | 495.0 | 50.0, 98.9 & 495.0 |

TABLE S4.
CLASSIFICATION OF THE 12 SUBSETS OF DATA (BEST AND WORST FITTING OF EACH) GIVEN IN SUPPORTING INFORMATION 2 ACCORDING WITH THE CLASSIFICATION SYSTEM PROPOSED IN THIS ARTICLE.

| **Subset** | **Tail** | **Head** | **Subset** | **Tail** | **Head** | **Subset** | **Tail** | **Head** |
|---|---|---|---|---|---|---|---|---|
| 1 (best) | D0 | A0 | 5 (best) | C- | A- | 9 (best) | C+ | A0 |
| 1 (worst) | D0 | B+ | 5 (worst) | C- | A- | 9 (worst) | C+ | A0 |
| 2 (best) | D0 | B+ | 6 (best) | C+ | B0 | 10 (best) | C+ | A0 |
| 2 (worst) | D- | B- | 6 (worst) | C+ | B0 | 10 (worst) | C+ | A0 |
| 3 (best) | C+ | A0 | 7 (best) | C+ | A- | 11 (best) | C+ | A0 |
| 3 (worst) | C+ | A+ | 7 (worst) | C+ | A0 | 11 (worst) | C+ | A0 |
| 4 (best) | D- | A0 | 8 (best) | C+ | A0 | 12 (best) | C+ | A0 |
| 4 (worst) | C- | A- | 8 (worst) | C+ | A0 | 12 (worst) | C+ | A0 |

# III. SUPPORTING INFORMATION 3.

TABLE S5
PARAMETER VALUES FOR THE BEST AND THE WORST FITTINGS OBTAINED FROM SET 1.

| Best fitting with the 3P algorithm | | | |
|---|---|---|---|
| **Parameter** | **3P** | **NLS** | **3P-NLS** |
| $x$ (Ω) | 985.9799 | 975.456 | 976.576 |
| $y$ (Ω) | -152.746 | -145.0487 | -146.1085 |
| $r$ (Ω) | 923.7354 | 913.5387 | 914.9415 |
| $R_0$ (Ω) | 1896.9989 | 1877.406 | 1879.776 |
| $R_\infty$ (Ω) | 74.9608 | 73.506 | 73.376 |
| $\tau$ (s) | 3.51E-05 | 3.42E-05 | 3.43E-05 |
| $\alpha$ | 0.8942 | 0.8985 | 0.8979 |
| $f$c (kHz) | 4.5393 | 4.6509 | 4.6391 |
| **Worst fitting with the 3P algorithm** | | | |
| $x$ (Ω) | 1867.1046 | 1808.97 | 1810.5 |
| $y$ (Ω) | -896.8529 | -802.0665 | -804.8654 |
| $r$ (Ω) | 1879.7417 | 1802.7537 | 1805.7909 |
| $R_0$ (Ω) | 3519.0985 | 3423.47 | 3427 |
| $R_\infty$ (Ω) | 215.1108 | 194.47 | 194 |
| $\tau$ | 2.44E-05 | 2.07E-05 | 2.07E-05 |
| $\alpha$ | 0.6834 | 0.7065 | 0.7059 |
| $f$c (kHz) | 6.5139 | 7.6992 | 7.68 |

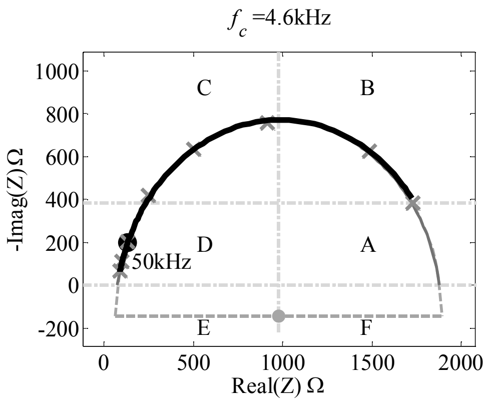


a)

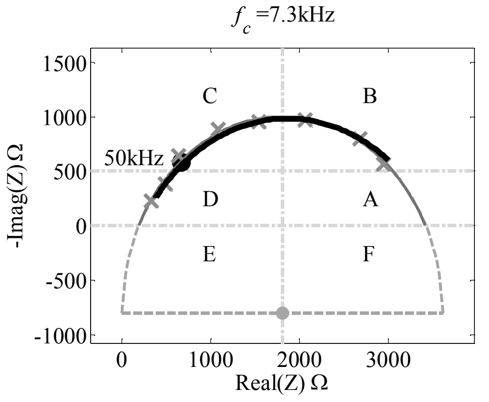


b)

Figure S1. Complex representation (Nyquist plot) of the best (a) and the worst (b) fittings obtained from set 1. The black, thicker line corresponds to the 3P fitting (it covers the frequencies of the spectra), the solid thinner line corresponds to the fitting with the NLS method (going from $R_0$ to $R_\infty$) and the dashed line corresponds to the 3P-NLS method (it covers the whole semicircle).

TABLE S6
PARAMETER VALUES FOR THE BEST AND THE WORST FITTINGS OBTAINED FROM SET 2.

| Parameter | 3P | NLS | 3P-NLS |
|---|---|---|---|
| **Best fitting with the 3P algorithm** | | | |
| $x$ (Ω) | 335.1432 | 321.668 | 324.644 |
| $y$ (Ω) | -111.865 | -100.0176 | -99.9216 |
| $r$ (Ω) | 338.8487 | 324.7315 | 324.6877 |
| $R_0$ (Ω) | 654.9942 | 630.613 | 633.574 |
| $R_\infty$ (Ω) | 15.2922 | 12.723 | 15.714 |
| $\tau$ (s) | 4.22E-05 | 3.83E-05 | 3.84E-05 |
| $\alpha$ | 0.7858 | 0.8007 | 0.8009 |
| $f$c (kHz) | 3.7704 | 4.1535 | 4.1448 |
| **Worst fitting with the 3P algorithm** | | | |
| $x$ (Ω) | 1013.2211 | 1025.058 | 1024.136 |
| $y$ (Ω) | -392.1576 | -461.6117 | -459.9188 |
| $r$ (Ω) | 983.651 | 1041.6211 | 1039.7507 |
| $R_0$ (Ω) | 1915.3195 | 1958.808 | 1956.636 |
| $R_\infty$ (Ω) | 111.1226 | 91.308 | 91.636 |
| $\tau$ | 2.25E-05 | 2.54E-05 | 2.53E-05 |
| $\alpha$ | 0.7389 | 0.7077 | 0.7083 |
| $f$c (kHz) | 7.0729 | 6.2714 | 6.2882 |

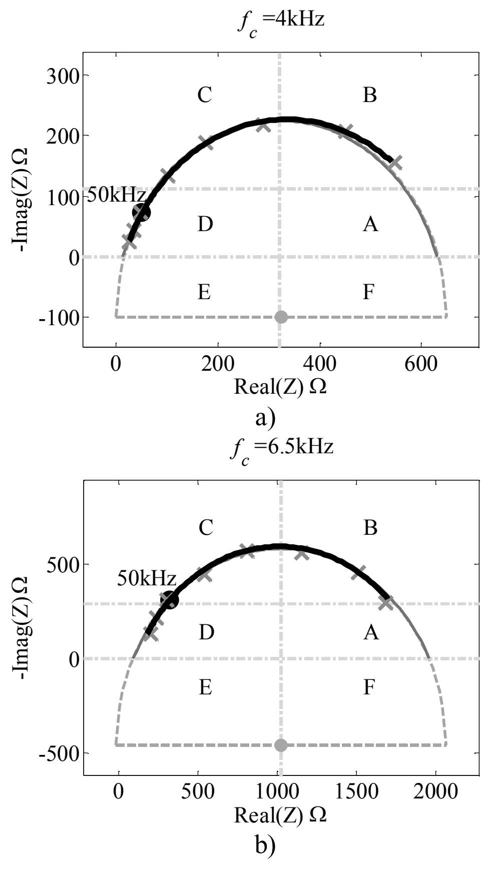


Figure S2. Complex representation (Nyquist plot) of the best (a) and the worst (b) fittings obtained from set 2. The black, thicker line corresponds to the 3P fitting (it covers the frequencies of the spectra), the solid thinner line corresponds to the fitting with the NLS method (going from $R_0$ to $R_\infty$) and the dashed line corresponds to the 3P-NLS method (it covers the whole semicircle).

TABLE S7
PARAMETER VALUES FOR THE BEST AND THE WORST FITTINGS OBTAINED FROM SET 3.

| Parameter | 3P | NLS | 3P-NLS |
|---|---|---|---|
| **Best fitting with the 3P algorithm** | | | |
| $x$ (Ω) | 288.6516 | 287.901 | 287.9015 |
| $y$ (Ω) | -10.1947 | -11.119 | -11.1197 |
| $r$ (Ω) | 24.964 | 26.071 | 26.0717 |
| $R_0$ (Ω) | 311.4391 | 311.482 | 311.483 |
| $R_\infty$ (Ω) | 265.864 | 264.32 | 264.32 |
| $\tau$ (s) | 2.22E-06 | 2.06E-06 | 2.06E-06 |
| $\alpha$ | 0.7322 | 0.7195 | 0.7195 |
| $f$c (kHz) | 71.8381 | 77.1686 | 77.1679 |
| **Worst fitting with the 3P algorithm** | | | |
| $x$ (Ω) | 563.9274 | 561.03 | 564.93 |
| $y$ (Ω) | -45.8128 | -59.4137 | -45.8289 |
| $r$ (Ω) | 106.3158 | 120.1483 | 106.3239 |
| $R_0$ (Ω) | 659.8661 | 665.46 | 660.87 |
| $R_\infty$ (Ω) | 467.9888 | 456.6 | 468.99 |
| $\tau$ | 3.55E-06 | 3.21E-06 | 3.55E-06 |
| $\alpha$ | 0.7164 | 0.6707 | 0.7163 |
| $f$c (kHz) | 44.777 | 49.5722 | 44.888 |

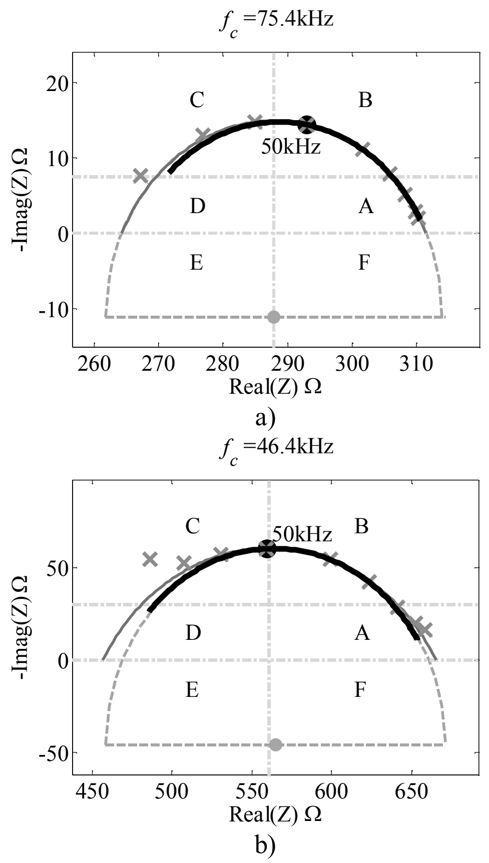


Figure S3. Complex representation (Nyquist plot) of the best (a) and the worst (b) fittings obtained from set 3. The black, thicker line corresponds to the 3P fitting (it covers the frequencies of the spectra), the solid thinner line corresponds to the fitting with the NLS method (going from $R_0$ to $R_\infty$) and the dashed line corresponds to the 3P-NLS method (it covers the whole semicircle).

TABLE S8
PARAMETER VALUES FOR THE BEST AND THE WORST FITTINGS OBTAINED FROM SET 4.

| Parameter | 3P | NLS | 3P-NLS |
|---|---|---|---|
| **Best fitting with the 3P algorithm** | | | |
| **Parameter** | **3P** | **NLS** | **3P-NLS** |
| $x$ (Ω) | 493.1263 | 492.51 | 493.125 |
| $y$ (Ω) | -31.5847 | -34.4381 | -31.5836 |
| $r$ (Ω) | 79.9055 | 82.565 | 79.9021 |
| $R_0$ (Ω) | 566.5245 | 567.55 | 566.52 |
| $R_\infty$ (Ω) | 419.7281 | 417.47 | 419.73 |
| $\tau$ (s) | 5.69E-06 | 5.52E-06 | 5.69E-06 |
| $\alpha$ | 0.7413 | 0.7281 | 0.7413 |
| $f$c (kHz) | 27.9546 | 28.8127 | 27.9579 |
| **Worst fitting with the 3P algorithm** | | | |
| $x$ (Ω) | 653.0328 | 647.77 | 653.035 |
| $y$ (Ω) | -56.5019 | -69.9399 | -56.5102 |
| $r$ (Ω) | 121.4071 | 134.1795 | 121.4172 |
| $R_0$ (Ω) | 760.4908 | 762.28 | 760.5 |
| $R_\infty$ (Ω) | 545.5748 | 533.26 | 545.57 |
| $\tau$ | 3.72E-06 | 3.22E-06 | 3.72E-06 |
| $\alpha$ | 0.6918 | 0.6635 | 0.6918 |
| $f$c (kHz) | 42.7852 | 49.4744 | 42.8018 |

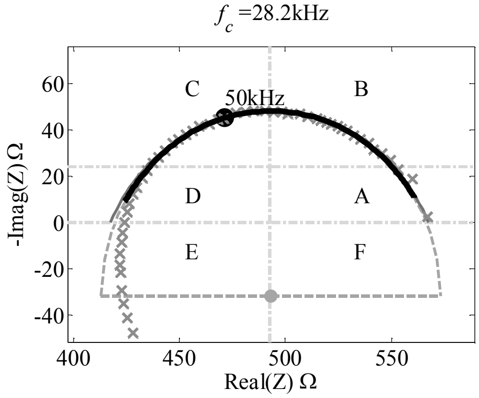


a)

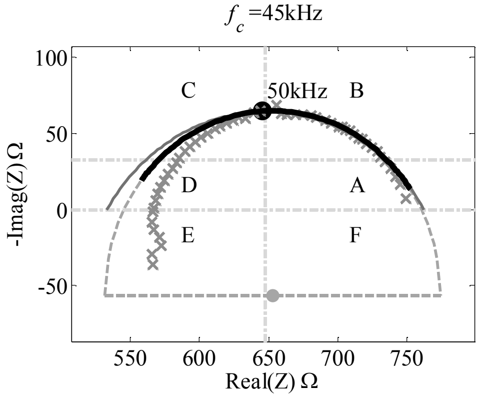


b)

Figure S4. Complex representation (Nyquist plot) of the best (a) and the worst (b) fittings obtained from set 4. The black, thicker line corresponds to the 3P fitting (it covers the frequencies of the spectra), the solid thinner line corresponds to the fitting with the NLS method (going from $R_0$ to $R_\infty$) and the dashed line corresponds to the 3P-NLS method (it covers the whole semicircle).

TABLE S9
PARAMETER VALUES FOR THE BEST AND THE WORST FITTINGS OBTAINED FROM SET 5.

| Parameter | 3P | NLS | 3P-NLS |
|---|---|---|---|
| **Best fitting with the 3P algorithm** | | | |
| $x$ (Ω) | 493.0965 | 490.8 | 492.1 |
| $y$ (Ω) | -37.9638 | -39.7977 | -38.4226 |
| $r$ (Ω) | 86.1282 | 87.7445 | 86.5376 |
| $R_0$ (Ω) | 570.4064 | 569.38 | 569.64 |
| $R_\infty$ (Ω) | 415.7866 | 412.6 | 414.56 |
| $\tau$ (s) | 4.48E-06 | 4.15E-06 | 4.28E-06 |
| $\alpha$ | 0.7094 | 0.7077 | 0.7103 |
| $f$c (kHz) | 35.4971 | 38.3802 | 37.2193 |
| **Worst fitting with the 3P algorithm** | | | |
| $x$ (Ω) | 665.9588 | 664.675 | 664.555 |
| $y$ (Ω) | -83.8775 | -71.0738 | -71.2351 |
| $r$ (Ω) | 150.8218 | 138.9312 | 139.0825 |
| $R_0$ (Ω) | 791.3054 | 784.05 | 784.01 |
| $R_\infty$ (Ω) | 540.6123 | 545.3 | 545.1 |
| $\tau$ | 3.50E-06 | 3.27E-06 | 3.26E-06 |
| $\alpha$ | 0.6246 | 0.6611 | 0.6609 |
| $f$c (kHz) | 45.5026 | 48.6534 | 48.7623 |

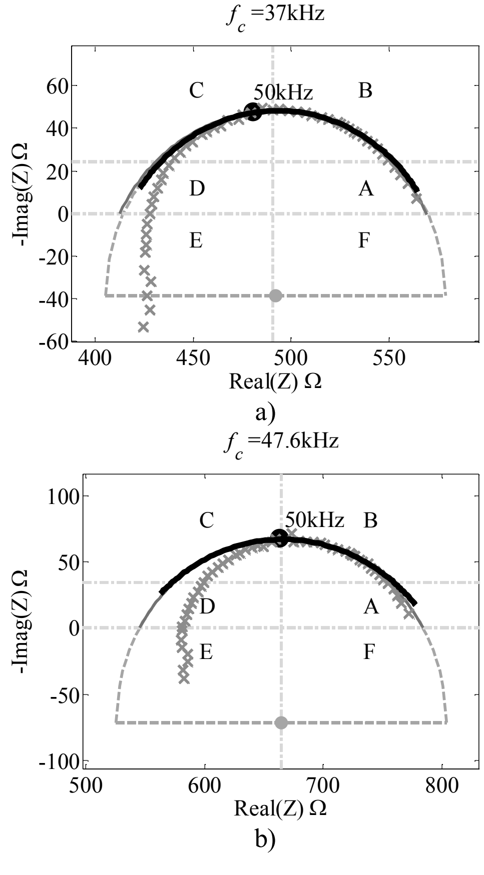


Figure S5. Complex representation (Nyquist plot) of the best (a) and the worst (b) fittings obtained from set 5. The black, thicker line corresponds to the 3P fitting (it covers the frequencies of the spectra), the solid thinner line corresponds to the fitting with the NLS method (going from $R_0$ to $R_\infty$) and the dashed line corresponds to the 3P-NLS method (it covers the whole semicircle).

TABLE S10

PARAMETER VALUES FOR THE BEST AND THE WORST FITTINGS OBTAINED FROM SET 6.

| Best fitting with the 3P algorithm | | | |
|---|---|---|---|
| **Parameter** | **3P** | **NLS** | **3P-NLS** |
| $x$ (Ω) | 471.2691 | 470.665 | 470.335 |
| $y$ (Ω) | -52.1943 | -49.0113 | -49.438 |
| $r$ (Ω) | 108.4563 | 105.4207 | 105.6198 |
| $R_0$ (Ω) | 566.3403 | 563.53 | 563.67 |
| $R_\infty$ (Ω) | 376.198 | 377.33 | 377 |
| $\tau$ (s) | 4.40E-06 | 4.34E-06 | 4.33E-06 |
| $\alpha$ | 0.6804 | 0.6939 | 0.6925 |
| $f$c (kHz) | 36.1726 | 36.6812 | 36.7915 |
| **Worst fitting with the 3P algorithm** | | | |
| $x$ (Ω) | 603.55 | 591.11 | 591.715 |
| $y$ (Ω) | -51.1916 | -102.5827 | -100.3892 |
| $r$ (Ω) | 109.4425 | 158.3579 | 156.236 |
| $R_0$ (Ω) | 700.282 | 711.75 | 711.43 |
| $R_\infty$ (Ω) | 506.8179 | 470.47 | 472 |
| $\tau$ | 3.43E-06 | 2.54E-06 | 2.58E-06 |
| $\alpha$ | 0.6901 | 0.5819 | 0.585 |
| $f$c (kHz) | 46.4107 | 62.7207 | 61.7258 |

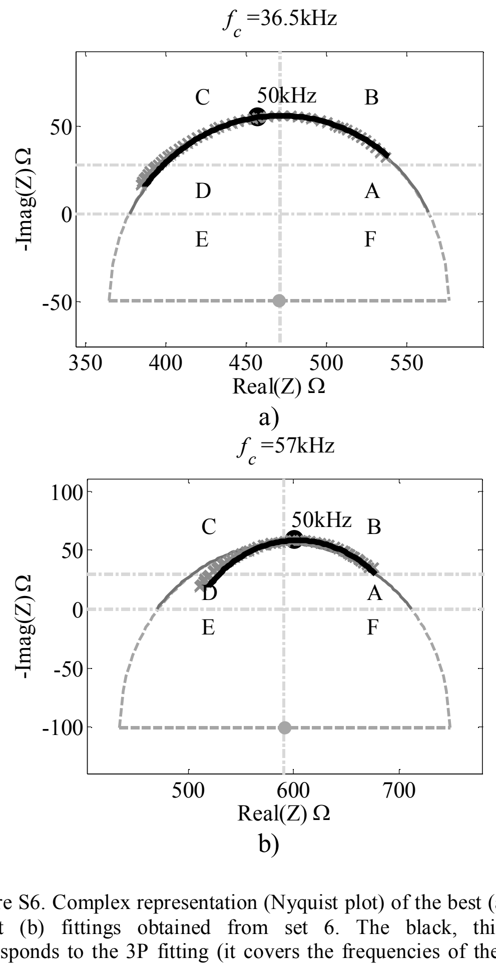


Figure S6. Complex representation (Nyquist plot) of the best (a) and the worst (b) fittings obtained from set 6. The black, thicker line corresponds to the 3P fitting (it covers the frequencies of the spectra), the solid thinner line corresponds to the fitting with the NLS method (going from $R_0$ to $R_\infty$) and the dashed line corresponds to the 3P-NLS method (it covers the whole semicircle).

TABLE S11
PARAMETER VALUES FOR THE BEST AND THE WORST FITTINGS OBTAINED FROM SET 7.

| Best fitting with the 3P algorithm | | | |
|---|---|---|---|
| **Parameter** | **3P** | **NLS** | **3P-NLS** |
| $x$ (Ω) | 33.1688 | 35.6785 | 35.656 |
| $y$ (Ω) | -11.7064 | -5.839 | -5.9218 |
| $r$ (Ω) | 17.8188 | 11.5122 | 11.609 |
| $R_0$ (Ω) | 46.6027 | 45.627 | 45.641 |
| $R_\infty$ (Ω) | 19.735 | 25.757 | 25.671 |
| $\tau$ (s) | 1.30E-07 | 2.53E-07 | 2.50E-07 |
| $\alpha$ | 0.5437 | 0.6614 | 0.6592 |
| $f$c (kHz) | 1220.7134 | 630.2837 | 636.3853 |
| **Worst fitting with the 3P algorithm** | | | |
| $x$ (Ω) | 31.2999 | 31.3695 | 30.6625 |
| $y$ (Ω) | -7.0558 | -6.8398 | -8.1201 |
| $r$ (Ω) | 12.13 | 11.8906 | 13.3365 |
| $R_0$ (Ω) | 41.1666 | 41.096 | 41.242 |
| $R_\infty$ (Ω) | 21.4332 | 21.643 | 20.083 |
| $\tau$ | 2.01E-07 | 2.06E-07 | 1.69E-07 |
| $\alpha$ | 0.6048 | 0.6098 | 0.5833 |
| $f$c (kHz) | 792.9475 | 771.3304 | 943.5474 |

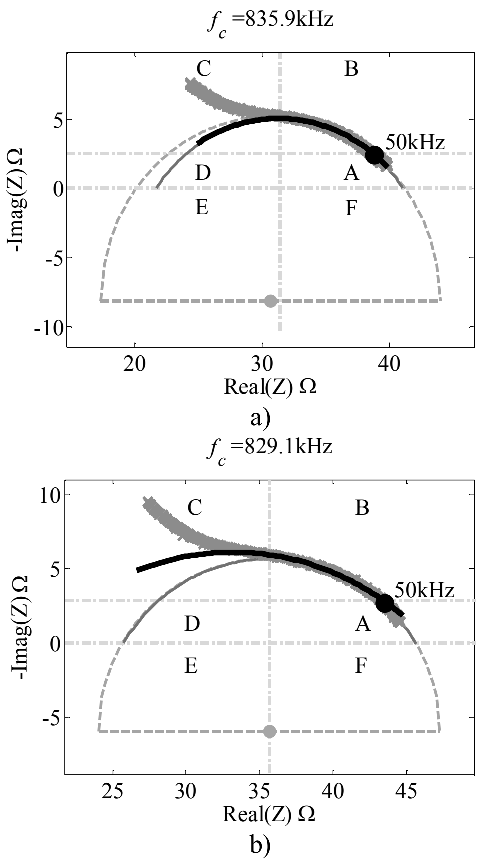


Figure S7. Complex representation (Nyquist plot) of the best (a) and the worst (b) fittings obtained from set 7. The black, thicker line corresponds to the 3P fitting (it covers the frequencies of the spectra), the solid thinner line corresponds to the fitting with the NLS method (going from $R_0$ to $R_\infty$) and the dashed line corresponds to the 3P-NLS method (it covers the whole semicircle).

TABLE S12
PARAMETER VALUES FOR THE BEST AND THE WORST FITTINGS OBTAINED FROM SET 8.

| Parameter | 3P | NLS | 3P-NLS |
|---|---|---|---|
| **Best fitting with the 3P algorithm** | | | |
| $x$ (Ω) | 397.4832 | 396.875 | 396.76 |
| $y$ (Ω) | -39.7585 | -42.3014 | -42.1049 |
| $r$ (Ω) | 99.9023 | 102.2823 | 101.9871 |
| $R_0$ (Ω) | 489.1332 | 489.69 | 489.65 |
| $R_\infty$ (Ω) | 305.8331 | 303.75 | 303.87 |
| $\tau$ (s) | 5.75E-07 | 5.72E-07 | 5.73E-07 |
| $\alpha$ | 0.7394 | 0.7286 | 0.7291 |
| $f$c (kHz) | 276.6514 | 278.031 | 277.7735 |
| **Worst fitting with the 3P algorithm** | | | |
| $x$ (Ω) | 398.1923 | 396.37 | 396.41 |
| $y$ (Ω) | -34.343 | -42.5552 | -42.418 |
| $r$ (Ω) | 95.1445 | 102.383 | 102.2533 |
| $R_0$ (Ω) | 486.9225 | 489.49 | 489.45 |
| $R_\infty$ (Ω) | 309.4621 | 303.25 | 303.37 |
| $\tau$ | 5.81E-07 | 5.67E-07 | 5.68E-07 |
| $\alpha$ | 0.7649 | 0.7271 | 0.7277 |
| $f$c (kHz) | 273.7526 | 280.5809 | 280.3348 |

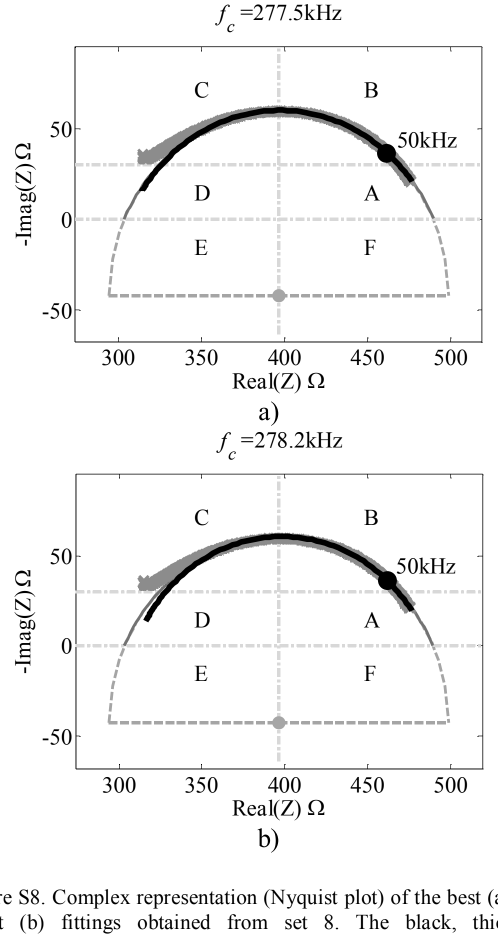


Figure S8. Complex representation (Nyquist plot) of the best (a) and the worst (b) fittings obtained from set 8. The black, thicker line corresponds to the 3P fitting (it covers the frequencies of the spectra), the solid thinner line corresponds to the fitting with the NLS method (going from $R_0$ to $R_\infty$) and the dashed line corresponds to the 3P-NLS method (it covers the whole semicircle).

TABLE S13

PARAMETER VALUES FOR THE BEST AND THE WORST FITTINGS OBTAINED FROM SET 9.

| Parameter | 3P | NLS | 3P-NLS |
|---|---|---|---|
| **Best fitting with the 3P algorithm** | | | |
| $x$ (Ω) | 297.4973 | 296.44 | 296.3145 |
| $y$ (Ω) | -18.3953 | -22.7077 | -22.6244 |
| $r$ (Ω) | 43.1448 | 47.359 | 47.2001 |
| $R_0$ (Ω) | 336.5241 | 337.748 | 337.739 |
| $R_\infty$ (Ω) | 258.4706 | 254.88 | 254.89 |
| $\tau$ (s) | 4.90E-07 | 4.66E-07 | 4.67E-07 |
| $\alpha$ | 0.7196 | 0.6817 | 0.6818 |
| $f$c (kHz) | 324.568 | 341.2595 | 341.1125 |
| **Worst fitting with the 3P algorithm** | | | |
| $x$ (Ω) | 170.3518 | 170.113 | 170.1315 |
| $y$ (Ω) | -14.0617 | -17.472 | -17.4053 |
| $r$ (Ω) | 36.3228 | 39.3541 | 39.2874 |
| $R_0$ (Ω) | 203.8423 | 205.375 | 205.353 |
| $R_\infty$ (Ω) | 136.8613 | 134.85 | 134.91 |
| $\tau$ | 6.63E-07 | 6.73E-07 | 6.73E-07 |
| $\alpha$ | 0.7469 | 0.7071 | 0.7078 |
| $f$c (kHz) | 239.9958 | 236.6344 | 236.3609 |

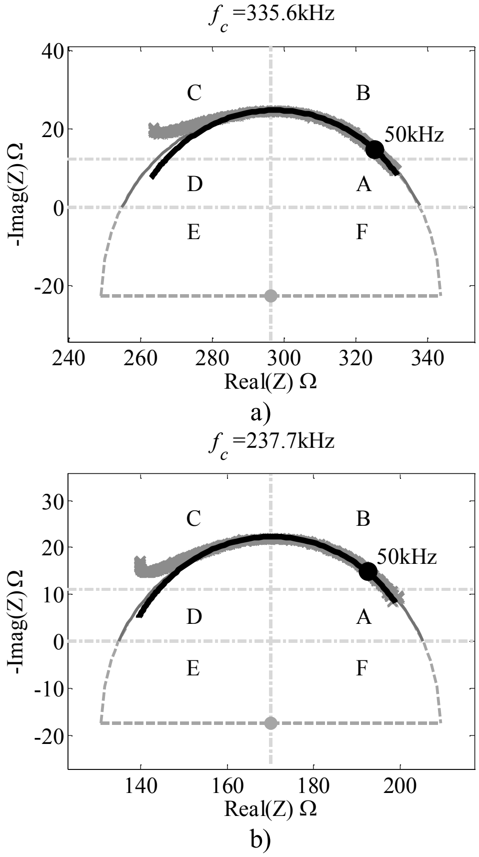


Figure S9. Complex representation (Nyquist plot) of the best (a) and the worst (b) fittings obtained from set 9. The black, thicker line corresponds to the 3P fitting (it covers the frequencies of the spectra), the solid thinner line corresponds to the fitting with the NLS method (going from $R_0$ to $R_\infty$) and the dashed line corresponds to the 3P-NLS method (it covers the whole semicircle).

TABLE S14
PARAMETER VALUES FOR THE BEST AND THE WORST FITTINGS OBTAINED FROM SET 10.

| Best fitting with the 3P algorithm | | | |
|---|---|---|---|
| **Parameter** | **3P** | **NLS** | **3P-NLS** |
| $x$ (Ω) | 394.7939 | 394.62 | 394.485 |
| $y$ (Ω) | -33.4571 | -35.4353 | -35.3832 |
| $r$ (Ω) | 89.8168 | 91.5185 | 91.3739 |
| $R_0$ (Ω) | 478.1465 | 478.72 | 478.73 |
| $R_\infty$ (Ω) | 311.4412 | 310.24 | 310.24 |
| $\tau$ (s) | 7.25E-07 | 7.26E-07 | 7.26E-07 |
| $\alpha$ | 0.757 | 0.7469 | 0.7469 |
| $f$c (kHz) | 219.4887 | 219.1032 | 219.0962 |
| **Worst fitting with the 3P algorithm** | | | |
| $x$ (Ω) | 424.9428 | 424.695 | 424.71 |
| $y$ (Ω) | -39.4754 | -41.8962 | -41.8267 |
| $r$ (Ω) | 100.2912 | 102.1975 | 102.128 |
| $R_0$ (Ω) | 517.1383 | 517.91 | 517.88 |
| $R_\infty$ (Ω) | 332.7472 | 331.48 | 331.54 |
| $\tau$ | 6.87E-07 | 6.93E-07 | 6.93E-07 |
| $\alpha$ | 0.7425 | 0.7311 | 0.7314 |
| $f$c (kHz) | 231.768 | 229.7235 | 229.6552 |

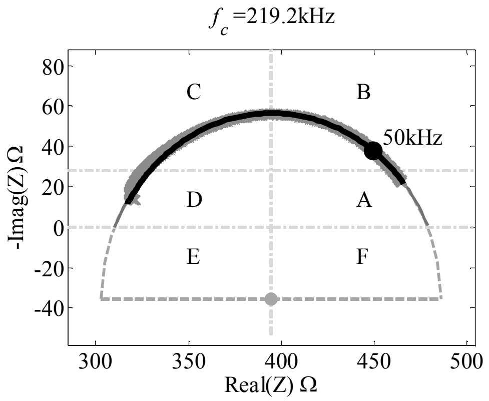


a)

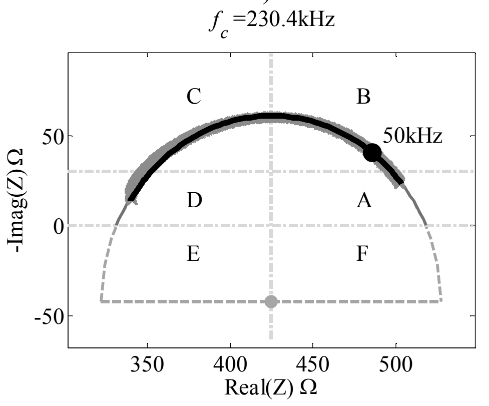


b)

Figure S10. Complex representation (Nyquist plot) of the best (a) and the worst (b) fittings obtained from set 10. The black, thicker line corresponds to the 3P fitting (it covers the frequencies of the spectra), the solid thinner line corresponds to the fitting with the NLS method (going from $R_0$ to $R_\infty$) and the dashed line corresponds to the 3P-NLS method (it covers the whole semicircle).

TABLE S15
PARAMETER VALUES FOR THE BEST AND THE WORST FITTINGS OBTAINED FROM SET 11.

| **Best fitting with the 3P algorithm** | | | |
|---|---|---|---|
| **Parameter** | **3P** | **NLS** | **3P-NLS** |
| $x$ (Ω) | 567.8832 | 568.075 | 567.98 |
| $y$ (Ω) | -43.4209 | -43.1332 | -43.1048 |
| $r$ (Ω) | 111.195 | 110.676 | 110.5867 |
| $R_0$ (Ω) | 670.2499 | 669.82 | 669.82 |
| $R_\infty$ (Ω) | 465.5165 | 466.15 | 466.14 |
| $\tau$ (s) | 7.20E-07 | 7.26E-07 | 7.26E-07 |
| $\alpha$ | 0.7446 | 0.7451 | 0.7451 |
| $f$c (kHz) | 220.9793 | 219.2917 | 219.3312 |
| **Worst fitting with the 3P algorithm** | | | |
| $x$ (Ω) | 419.5404 | 421.74 | 421.75 |
| $y$ (Ω) | -30.8442 | -21.801 | -21.6134 |
| $r$ (Ω) | 83.9065 | 75.3909 | 75.2315 |
| $R_0$ (Ω) | 497.572 | 493.91 | 493.81 |
| $R_\infty$ (Ω) | 341.5088 | 349.57 | 349.69 |
| $\tau$ | 8.30E-07 | 8.73E-07 | 8.74E-07 |
| $\alpha$ | 0.7604 | 0.8132 | 0.8145 |
| $f$c (kHz) | 191.764 | 182.3024 | 182.1947 |

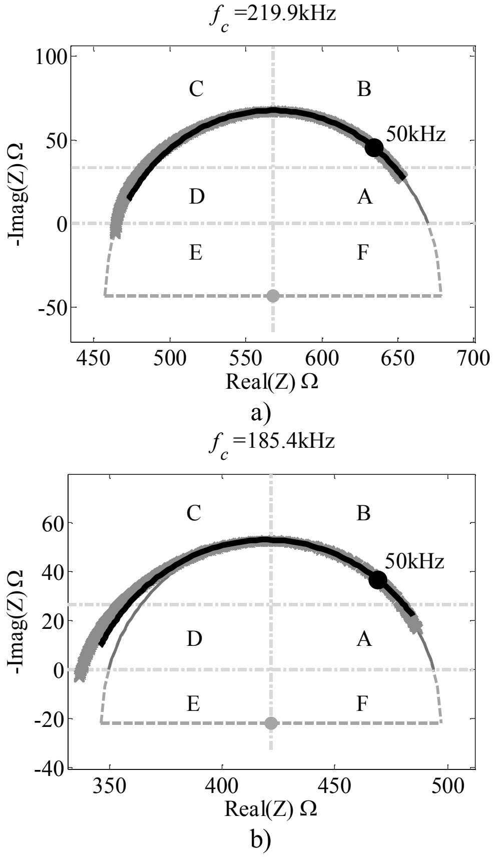


Figure S11. Complex representation (Nyquist plot) of the best (a) and the worst (b) fittings obtained from set 11. The black, thicker line corresponds to the 3P fitting (it covers the frequencies of the spectra), the solid thinner line corresponds to the fitting with the NLS method (going from $R_0$ to $R_\infty$) and the dashed line corresponds to the 3P-NLS method (it covers the whole semicircle).

TABLE S16

PARAMETER VALUES FOR THE BEST AND THE WORST FITTINGS OBTAINED FROM SET 12.

| Parameter | 3P | NLS | 3P-NLS |
|---|---|---|---|
| **Best fitting with the 3P algorithm** | | | |
| $x$ (Ω) | 538.3024 | 538.765 | 538.64 |
| $y$ (Ω) | -41.5563 | -38.1459 | -38.1496 |
| $r$ (Ω) | 98.5322 | 95.2106 | 95.1342 |
| $R_0$ (Ω) | 627.6426 | 625.77 | 625.79 |
| $R_\infty$ (Ω) | 448.9622 | 451.53 | 451.49 |
| $\tau$ (s) | 7.50E-07 | 7.59E-07 | 7.58E-07 |
| $\alpha$ | 0.7228 | 0.7376 | 0.7373 |
| $f$c (kHz) | 212.3348 | 209.7773 | 209.8574 |
| **Worst fitting with the 3P algorithm** | | | |
| $x$ (Ω) | 538.1589 | 538.04 | 538.065 |
| $y$ (Ω) | -35.9861 | -38.8042 | -38.7108 |
| $r$ (Ω) | 93.5845 | 95.6727 | 95.5846 |
| $R_0$ (Ω) | 624.5478 | 625.49 | 625.46 |
| $R_\infty$ (Ω) | 451.77 | 450.59 | 450.67 |
| $\tau$ | 7.46E-07 | 7.50E-07 | 7.50E-07 |
| $\alpha$ | 0.7487 | 0.7341 | 0.7346 |
| $f$c (kHz) | 213.3333 | 212.2026 | 212.0713 |

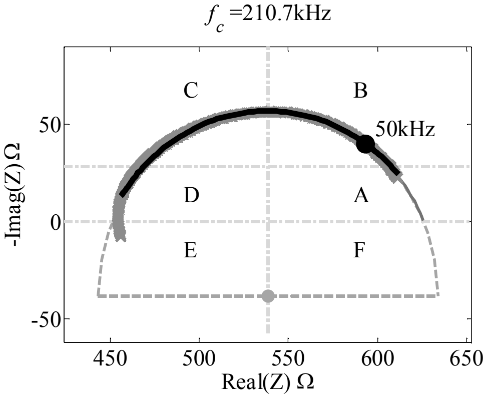


a)

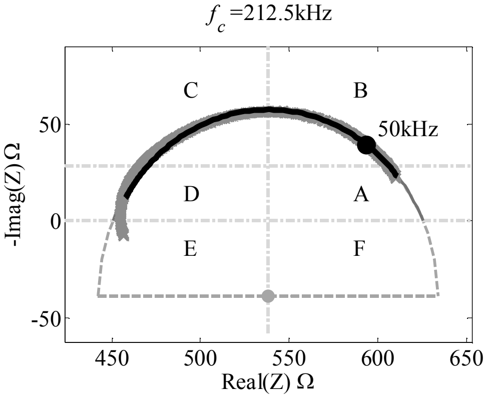


b)

Figure S12. Complex representation (Nyquist plot) of the best (a) and the worst (b) fittings obtained from set 12. The black, thicker line corresponds to the 3P fitting (it covers the frequencies of the spectra), the solid thinner line corresponds to the fitting with the NLS method (going from $R_0$ to $R_\infty$) and the dashed line corresponds to the 3P-NLS method (it covers the whole semicircle).

## IV. SUPPORTING INFORMATION 4.

In order to obtain the average percentage of the residuals given after the fitting performed (%SS and %SSR), we used the next expressions.

$$\%SS = \sqrt{\frac{SS}{N}} x100$$

$$\%SSR = \sqrt{\frac{SSR}{N}} x100$$

Where:

$$SS: sum\ of\ squares$$
$$SSR: sum\ of\ squares\ radial$$
$$N: number\ of\ impedance\ points$$

## V. SUPPORTING INFORMATION 5

TABLE S17.
PARAMETERS OBTAINED BY THE 3P METHOD WITH SIMULATED SPECTRA CREATED WITH DIFFERENT PERCENTAGES OF ADDED NOISE. FORM 1.

| Parameter | Noise level (percent) | | | | Theoretical value |
|---|---|---|---|---|---|
| | 1% | 2% | 5% | 10% | |
| Mean $R_0$ | 500.86 | 499.84 | 502.59 | 512.57 | 500 |
| Mean $R_{inf}$ | 249.77 | 250.02 | 252.19 | 248.87 | 250 |
| Mean $\tau$ | 5.66E-06 | 5.63E-06 | 5.73E-06 | 6.35E-06 | 5.60E-06 |
| Mean $\alpha$ | 0.7977 | 0.8016 | 0.7978 | 0.7811 | 0.8 |
| SD $R_0$ | 3.54 | 6.93 | 7.95 | 16.56 | |
| SD $R_{inf}$ | 2.26 | 4.67 | 5.03 | 9.34 | |
| SD $\tau$ | 1.90E-07 | 3.93E-07 | 4.69E-07 | 7.18E-07 | |
| SD $\alpha$ | 1.47E-02 | 2.96E-02 | 3.23E-02 | 5.37E-02 | |
| No. Spec. | 100 | 100 | 49 | 40 | |

TABLE S18.
PARAMETERS OBTAINED BY THE 3P METHOD WITH SIMULATED SPECTRA CREATED WITH DIFFERENT PERCENTAGES OF ADDED NOISE. FORM 2.

| First half (lower frequencies) | | | | | |
|---|---|---|---|---|---|
| **Parameter** | **Noise level (Percent)** | | | | **Theoretical value** |
| | 1% | 2% | 5% | 10% | |
| **Mean** $R_0$ | 501.26 | 500.55 | 510.75 | 527.58 | 500 |
| **Mean** $R_{inf}$ | 246.19 | 243.63 | 218.14 | 219.60 | 250 |
| **Mean** $\tau$ | 5.55E-06 | 5.47E-06 | 4.89E-06 | 5.50E-06 | 5.60E-06 |
| **Mean** $\alpha$ | 0.7903 | 0.7935 | 0.7202 | 0.7099 | 0.8 |
| **SD** $R_0$ | 4.83 | 8.92 | 6.78 | 10.03 | |
| **SD** $R_{inf}$ | 15.52 | 27.06 | 23.52 | 26.56 | |
| **SD** $\tau$ | 4.42E-07 | 7.51E-07 | 7.42E-07 | 9.44E-07 | |
| **SD** $\alpha$ | 4.84E-02 | 8.32E-02 | 5.71E-02 | 7.49E-02 | |
| **No. Spec.** | 100 | 98 | 38 | 18 | |
| **Second half (higher frequencies)** | | | | | |
| **Parameter** | **Noise level (Percent)** | | | | **Theoretical value** |
| | 1% | 2% | 5% | 10% | |
| **Mean** $R_0$ | 500.42 | 505.22 | 527.86 | 550.20 | 500 |
| **Mean** $R_{inf}$ | 249.98 | 249.71 | 244.87 | 238.43 | 250 |
| **Mean** $\tau$ | 5.71E-06 | 6.15E-06 | 7.88E-06 | 1.09E-05 | 5.60E-06 |
| **Mean** $\alpha$ | 0.8006 | 0.7959 | 0.7399 | 0.6919 | 0.8 |
| **SD** $R_0$ | 13.27 | 26.22 | 26.06 | 44.16 | |
| **SD** $R_{inf}$ | 2.60 | 4.77 | 4.96 | 7.95 | |
| **SD** $\tau$ | 8.47E-07 | 1.88E-06 | 2.21E-06 | 5.05E-06 | |
| **SD** $\alpha$ | 3.51E-02 | 6.38E-02 | 5.37E-02 | 6.43E-02 | |
| **No. Spec.** | 100 | 100 | 41 | 30 | |

TABLE S19.
PARAMETERS OBTAINED BY THE 3P METHOD WITH SIMULATED SPECTRA CREATED WITH DIFFERENT PERCENTAGES OF ADDED NOISE. FORM 3.

| First third (lower frequencies) | | | | | |
|---|---|---|---|---|---|
| **Parameter** | **Noise level (Percent)** | | | | **Theoretical value** |
| | 1% | 2% | 5% | 10% | |
| **Mean** $R_0$ | 500.54 | 501.51 | 517.67 | 541.93 | 500 |
| **Mean** $R_{inf}$ | 254.45 | 237.63 | 187.69 | 298.94 | 250 |
| **Mean** $\tau$ | 5.99E-06 | 5.50E-06 | 4.09E-06 | 1.09E-05 | 5.60E-06 |
| **Mean** $\alpha$ | 0.8076 | 0.7891 | 0.6634 | 0.7158 | 0.8 |
| **SD** $R_0$ | 2.90 | 4.55 | 8.11 | 8.35 | |
| **SD** $R_{inf}$ | 29.97 | 44.62 | 45.68 | 45.59 | |
| **SD** $\tau$ | 1.20E-06 | 1.51E-06 | 1.40E-06 | 2.20E-06 | |
| **SD** $\alpha$ | 4.12E-02 | 4.85E-02 | 8.08E-02 | 1.49E-01 | |
| **No. Spec.** | 30 | 21 | 7 | 6 | |
| **Middle third** | | | | | |
| **Parameter** | **Noise level (Percent)** | | | | **Theoretical value** |
| | 1% | 2% | 5% | 10% | |
| **Mean** $R_0$ | 499.30 | 509.49 | 533.34 | 559.95 | 500 |
| **Mean** $R_{inf}$ | 250.85 | 241.85 | 223.23 | 216.46 | 250 |
| **Mean** $\tau$ | 5.62E-06 | 5.76E-06 | 5.97E-06 | 6.88E-06 | 5.60E-06 |
| **Mean** $\alpha$ | 0.8054 | 0.7599 | 0.6801 | 0.6298 | 0.8 |
| **SD** $R_0$ | 6.58 | 9.52 | 25.13 | 30.65 | |
| **SD** $R_{inf}$ | 5.48 | 8.41 | 21.52 | 22.47 | |
| **SD** $\tau$ | 1.07E-07 | 1.39E-07 | 3.78E-07 | 6.46E-07 | |
| **SD** $\alpha$ | 3.13E-02 | 4.11E-02 | 1.03E-01 | 9.99E-02 | |
| **No. Spec.** | 50 | 44 | 28 | 20 | |
| **Last third (higher frequencies)** | | | | | |
| **Parameter** | **Noise level (Percent)** | | | | **Theoretical value** |
| | 1% | 2% | 5% | 10% | |
| **Mean** $R_0$ | 470.89 | 465.64 | 400.42 | 647.57 | 500 |
| **Mean** $R_{inf}$ | 250.47 | 250.71 | 246.45 | 215.70 | 250 |
| **Mean** $\tau$ | 4.68E-06 | 4.29E-06 | 2.66E-06 | 7.62E-04 | 5.60E-06 |
| **Mean** $\alpha$ | 0.8372 | 0.8540 | 0.8430 | 0.4812 | 0.8 |
| **SD** $R_0$ | 64.49 | 48.47 | 4.39 | 2.37 | |
| **SD** $R_{inf}$ | 1.03 | 1.44 | 3.04 | 0.08 | |
| **SD** $\tau$ | 2.61E-06 | 1.64E-06 | 7.20E-07 | 1.29E-05 | |
| **SD** $\alpha$ | 4.42E-02 | 4.79E-02 | 8.69E-02 | 1.52E-03 | |
| **No. Spec.** | 18 | 11 | 5 | 3 | |

# VI. Supporting information 6

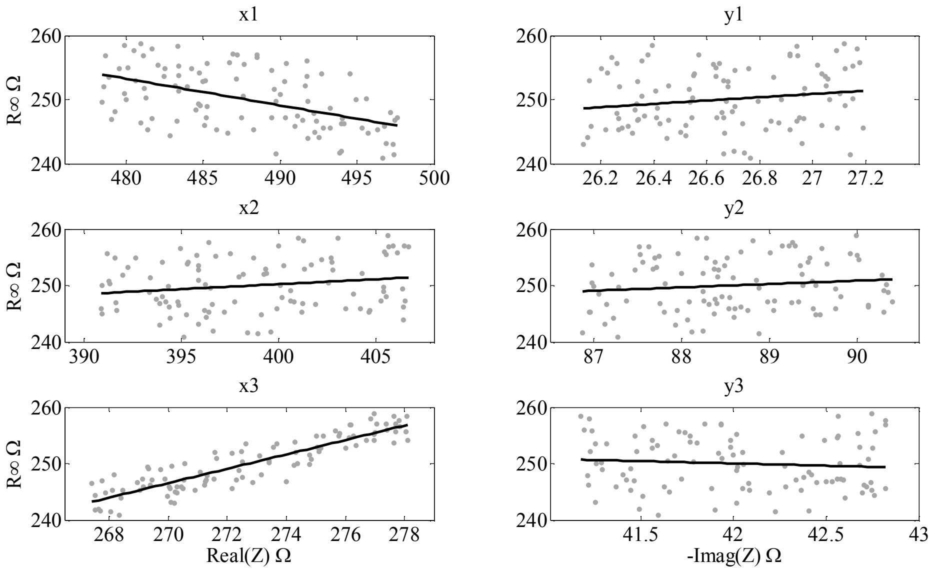


**Figure S13**. Scatter plots for parameter $R_\infty$, at 2% of noise, Form 1.

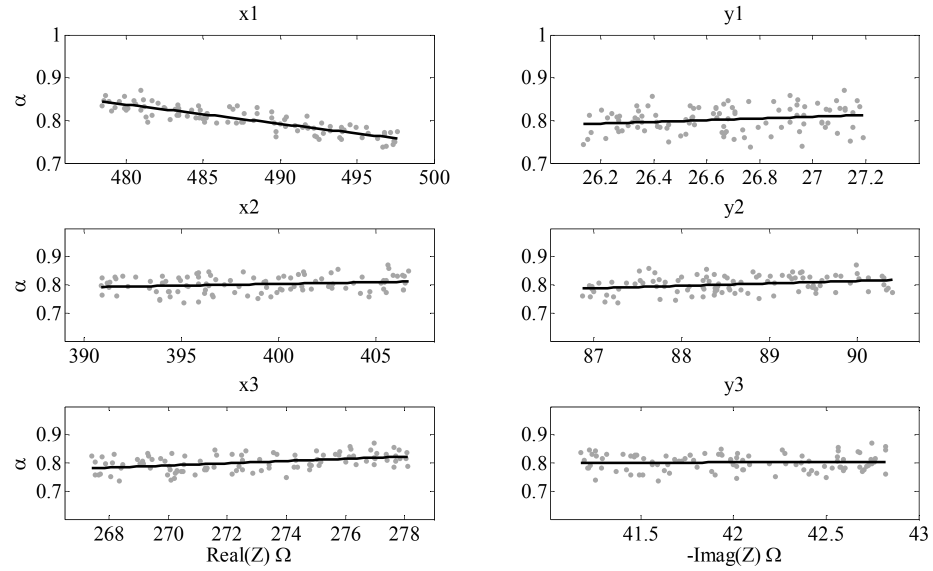


**Figure S14**. Scatter plots for parameter $\alpha$, at 2% of noise, Form 1.

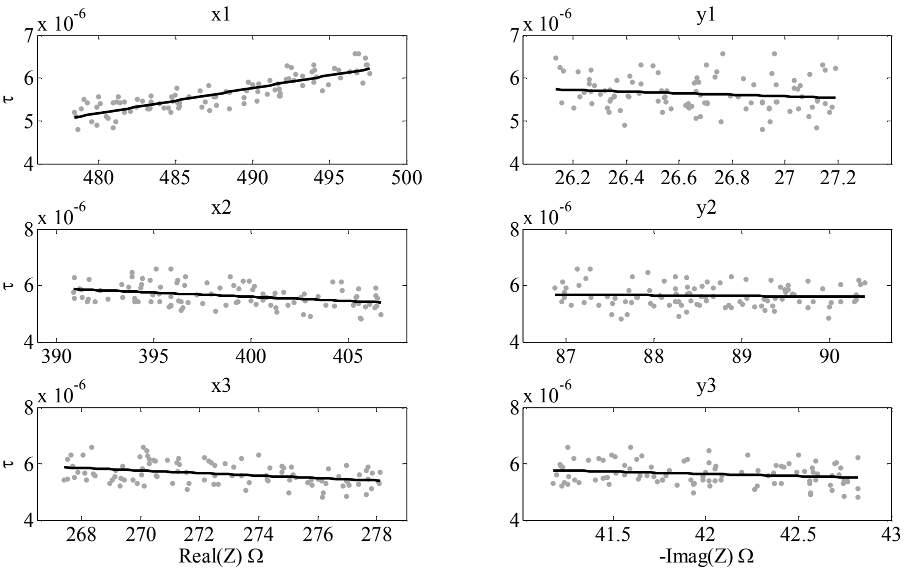


**Figure S15**. Scatter plots for parameter $\tau$, at 2% of noise, Form 1

In the **Tables S20** to **Table S25**, NL: Noise Level, SP: Scatter Plot linear parameters, *m* is the slope, *b* is the y-intercept and r is de Pearson correlation coefficient of the linear fit for the data, *x*i: abscise i, *y*i: ordinate i. The higher values of *r* are shown in bold.

TABLE S20.
LINEAR RELATIONSHIPS BETWEEN PARAMETERS AND COORDINATE POINTS BY SCATTER PLOTS ANALYSIS.

| | | | **Form 1** | | | | | |
|---|---|---|---|---|---|---|---|---|
| | **NL** | **SP** | **x1** | **y1** | **x2** | **y2** | **x3** | **y3** |
| $R_0$ | **1%** | **m** | 1.2057 | 1.402 | -0.0090732 | -0.64021 | -0.25581 | 0.55789 |
| | | **b** | -88.317 | 463.48 | 504.48 | 557.64 | 570.63 | 477.43 |
| | | **r** | **0.99516** | 0.06574 | -0.0057085 | -0.085819 | -0.11798 | 0.038985 |
| | **2%** | **m** | 1.2005 | -4.1512 | 0.019462 | -0.71053 | -0.44242 | -0.48297 |
| | | **b** | -85.76 | 610.52 | 492.07 | 562.78 | 620.5 | 520.13 |
| | | **r** | **0.99485** | -0.18392 | 0.013368 | -0.099962 | -0.21028 | -0.036241 |
| | **5%** | **m** | 1.231 | 1.2393 | -0.36175 | -0.62148 | -0.33721 | 0.2862 |
| | | **b** | -101.07 | 467.42 | 645.28 | 555.78 | 592.96 | 488.65 |
| | | **r** | **0.99102** | 0.054755 | -0.24973 | -0.095212 | -0.15325 | 0.021138 |
| | **10%** | **m** | 1.2317 | -0.71349 | -0.14369 | -0.22599 | -0.22205 | 0.77355 |
| | | **b** | -103.1 | 516.89 | 555.23 | 517.69 | 558.84 | 465.34 |
| | | **r** | **0.99252** | -0.028884 | -0.087854 | -0.031283 | -0.094609 | 0.052367 |
| $R_\infty$ | **1%** | **m** | -0.36658 | -0.58436 | -0.004231 | 1.1079 | 1.2195 | -1.414 |
| | | **b** | 428.9 | 265.35 | 251.46 | 151.52 | -82.836 | 309.17 |
| | | **r** | -0.47455 | -0.042977 | -0.0041751 | 0.23294 | **0.88218** | -0.15498 |
| | **2%** | **m** | -0.41667 | 2.5695 | 0.17552 | 0.59163 | 1.2763 | -0.80498 |
| | | **b** | 453.27 | 181.51 | 180.04 | 197.61 | -98.077 | 283.84 |
| | | **r** | -0.51192 | 0.16878 | 0.17874 | 0.1234 | **0.89938** | -0.089552 |
| | **5%** | **m** | -0.41454 | -1.945 | 0.31454 | 1.3172 | 1.2733 | 0.070662 |
| | | **b** | 453.84 | 303.44 | 125.49 | 134.48 | -97.116 | 248.21 |

| | | | | | | | | |
|---|---|---|---|---|---|---|---|---|
| | | r | -0.49523 | -0.12752 | 0.32222 | 0.29946 | **0.85868** | 0.0077445 |
| | **10%** | m | -0.3899 | 1.6467 | 0.10552 | 0.62504 | 1.2014 | -0.58044 |
| | | b | 445.98 | 211.6 | 213.57 | 200.63 | -74.792 | 280.1 |
| | | r | -0.5097 | 0.10814 | 0.10466 | 0.14036 | **0.83043** | -0.063746 |
| $\alpha$ | **1%** | m | -0.0044635 | -0.0064216 | 0.00092704 | 0.0094663 | 0.0031066 | -0.0034389 |
| | | b | 2.9788 | 0.96888 | 0.42782 | -0.041787 | -0.049613 | 0.94211 |
| | | r | **-0.88445** | -0.072291 | 0.14003 | **0.30464** | **0.34398** | -0.057693 |
| | **2%** | m | -0.0044968 | 0.020118 | 0.0012142 | 0.0085985 | 0.0039307 | 0.00074733 |
| | | b | 2.9951 | 0.26522 | 0.31747 | 0.039922 | -0.27041 | 0.77024 |
| | | r | **-0.87196** | 0.20857 | 0.19516 | 0.28306 | 0.43716 | 0.013122 |
| | **5%** | m | -0.0050668 | -0.012314 | 0.0030918 | 0.011387 | 0.0040506 | -0.0026752 |
| | | b | 3.2885 | 1.1424 | -0.42404 | -0.19735 | -0.2965 | 0.92436 |
| | | r | **-0.83882** | -0.11188 | 0.43893 | 0.35876 | 0.37855 | -0.040632 |
| | **10%** | m | -0.0023795 | -0.0054103 | -0.00017613 | 0.0043851 | 0.0016899 | -0.0024136 |
| | | b | 1.9612 | 0.94563 | 0.87094 | 0.41353 | 0.3355 | 0.90159 |
| | | r | **-0.62062** | -0.070893 | -0.034856 | 0.19647 | 0.23305 | -0.052886 |
| $\tau$ | **1%** | m | 5.79E-08 | 1.12E-07 | -2.74E-08 | -3.34E-08 | -2.61E-08 | -9.39E-08 |
| | | b | -2.26E-05 | 2.68E-06 | 1.66E-05 | 8.62E-06 | 1.28E-05 | 9.61E-06 |
| | | r | **0.88995** | 0.097688 | -0.32158 | -0.083311 | -0.22393 | -0.12226 |
| | **2%** | m | 5.90E-08 | -1.86E-07 | -3.00E-08 | -2.42E-08 | -4.44E-08 | -1.59E-07 |
| | | b | -2.32E-05 | 1.06E-05 | 1.76E-05 | 7.78E-06 | 1.78E-05 | 1.23E-05 |
| | | r | **0.86268** | -0.14538 | -0.36306 | -0.06010 | -0.37251 | -0.21108 |
| | **5%** | m | 6.29E-08 | 5.40E-08 | -4.81E-08 | -4.86E-08 | -4.37E-08 | -1.24E-07 |
| | | b | -2.50E-05 | 4.26E-06 | 2.49E-05 | 1.00E-05 | 1.77E-05 | 1.09E-05 |
| | | r | **0.86022** | 0.040558 | -0.56396 | -0.12659 | -0.33759 | -0.15524 |
| | **10%** | m | 6.40E-08 | -1.93E-07 | -4.23E-08 | -5.39E-08 | -3.14E-08 | -1.66E-07 |
| | | b | -2.54E-05 | 1.10E-05 | 2.27E-05 | 1.06E-05 | 1.44E-05 | 1.27E-05 |
| | | r | **0.84155** | -0.12752 | -0.42248 | -0.12179 | -0.21831 | -0.18322 |

TABLE S21.
LINEAR RELATIONSHIPS BETWEEN PARAMETERS AND COORDINATE POINTS BY SCATTER PLOTS ANALYSIS.

| **Form 2 (First Middle).** | | | | | | | | |
|---|---|---|---|---|---|---|---|---|
| | **NL** | **SP** | **x1** | **y1** | **x2** | **y2** | **x3** | **y3** |
| $R_0$ | **1%** | m | 1.5777 | 1.9797 | -0.64625 | -1.3783 | 0.12285 | 1.4766 |
| | | b | -269.7 | 448.48 | 787.35 | 600.36 | 455.95 | 367.52 |
| | | r | **0.95512** | 0.06809 | -0.34467 | -0.12878 | 0.052149 | 0.14872 |
| | **2%** | m | 1.5439 | -5.3199 | -0.56094 | -1.1013 | 0.069622 | -0.06334 |
| | | b | -252.93 | 642.02 | 748.24 | 579.39 | 474.5 | 505.92 |
| | | r | **0.96164** | -0.17716 | -0.28463 | -0.10071 | 0.0309 | -0.0070799 |
| | **5%** | m | 1.5737 | -0.44797 | -0.54221 | 0.80088 | -0.32575 | -0.94696 |
| | | b | -268.41 | 512.63 | 741.07 | 443.02 | 620.74 | 586.45 |
| | | r | **0.94889** | -0.014849 | -0.29345 | 0.072387 | -0.14924 | -0.10642 |

| | | | | | | | | |
|---|---|---|---|---|---|---|---|---|
| | **10%** | **m** | 1.5527 | -13.018 | -1.377 | -0.27357 | 1.3261 | 2.8378 |
| | | **b** | -238.75 | 872.89 | 1146.7 | 544.37 | 38.084 | 268.73 |
| | | **r** | **0.33901** | -0.14626 | -0.23267 | -0.0086089 | 0.21215 | 0.1177 |
| $R_\infty$ | **1%** | **m** | -3.6252 | -5.9306 | 4.3555 | 8.6653 | -0.007215 | -7.0193 |
| | | **b** | 2017.7 | 404.32 | -1681.9 | -376.8 | 248.85 | 881.98 |
| | | **r** | **-0.68277** | -0.063457 | **0.72268** | 0.25188 | -0.00095283 | -0.21994 |
| | **2%** | **m** | -3.4319 | 12.29 | 3.9573 | 5.7626 | -0.58028 | -2.4705 |
| | | **b** | 1918.9 | -82.894 | -1505.2 | -169.74 | 458.74 | 468.94 |
| | | **r** | **-0.70282** | 0.13456 | **0.66019** | 0.17327 | -0.084674 | -0.090792 |
| | **5%** | **m** | -3.2016 | 10.295 | 4.0918 | 3.3722 | 1.2056 | 0.75017 |
| | | **b** | 1800.8 | -40.323 | -1578.5 | -6.079 | -208.35 | 168.3 |
| | | **r** | **-0.60652** | 0.10721 | **0.6958** | 0.095764 | 0.17354 | 0.026487 |
| | **10%** | **m** | -1.4077 | 1.2089 | -0.52736 | 4.5204 | 1.1706 | -0.36492 |
| | | **b** | 971.29 | 246.88 | 517.43 | -47.248 | -150.23 | 312.12 |
| | | **r** | **-0.37663** | 0.016644 | -0.10919 | 0.17431 | 0.22947 | -0.018547 |
| $\alpha$ | **1%** | **m** | -0.012736 | -0.018572 | 0.012429 | 0.023565 | -0.00064702 | -0.013858 |
| | | **b** | 7.0141 | 1.2855 | -4.7117 | -0.90386 | 1.029 | 2.0456 |
| | | **r** | **-0.76923** | -0.063725 | **0.6613** | 0.21966 | -0.027401 | -0.13925 |
| | **2%** | **m** | -0.011637 | 0.044519 | 0.011068 | 0.01701 | -0.0013119 | -0.00053017 |
| | | **b** | 6.4735 | -0.39026 | -4.0979 | -0.42683 | 1.2805 | 0.8449 |
| | | **r** | **-0.77947** | 0.15942 | **0.60394** | 0.16728 | -0.062612 | -0.0063726 |
| | **5%** | **m** | -0.0039364 | 0.019638 | 0.0044635 | -0.0013997 | 0.0015367 | 0.0049843 |
| | | **b** | 2.6664 | 0.21518 | -1.2368 | 0.84347 | 0.17607 | 0.29093 |
| | | **r** | **-0.39152** | 0.10737 | **0.39849** | -0.020868 | 0.11613 | 0.092395 |
| | **10%** | **m** | -0.0013159 | 0.022016 | 0.0017459 | 0.0061328 | -0.0024396 | -0.0070606 |
| | | **b** | 1.338 | 0.10208 | -0.097625 | 0.24813 | 1.5861 | 1.3277 |
| | | **r** | **-0.19811** | **0.17055** | **0.20341** | **0.13307** | **-0.2691** | **-0.20193** |
| $\tau$ | **1%** | **m** | -9.15E-08 | -1.06E-07 | 1.24E-07 | 3.24E-07 | -4.73E-08 | -2.36E-07 |
| | | **b** | 5.03E-05 | 8.37E-06 | -4.93E-05 | -1.77E-05 | 2.30E-05 | 2.69E-05 |
| | | **r** | **-0.60522** | -0.039817 | **0.72142** | 0.33026 | -0.21934 | -0.25961 |
| | **2%** | **m** | -7.89E-08 | 4.11E-07 | 1.13E-07 | 2.29E-07 | -6.18E-08 | -1.04E-07 |
| | | **b** | 4.40E-05 | -5.46E-06 | -4.46E-05 | -1.10E-05 | 2.83E-05 | 1.49E-05 |
| | | **r** | **-0.58767** | 0.1637 | **0.6881** | 0.25093 | -0.32803 | -0.13881 |
| | **5%** | **m** | 3.69E-08 | 2.20E-07 | 1.06E-08 | 3.60E-08 | -2.66E-08 | -1.04E-07 |
| | | **b** | -1.36E-05 | -1.44E-06 | -2.20E-07 | 1.88E-06 | 1.43E-05 | 1.39E-05 |
| | | **r** | **0.18935** | 0.061995 | 0.0487 | 0.027689 | -0.10364 | -0.09968 |
| | **10%** | **m** | -4.36E-06 | 4.43E-05 | 9.82E-06 | -1.63E-05 | -4.67E-06 | -9.19E-06 |
| | | **b** | 0.0020304 | -0.0012989 | -0.0045508 | 0.0010606 | 0.0015975 | 0.00071381 |
| | | **r** | **-0.19062** | 0.099456 | **0.33191** | **-0.10249** | **-0.14939** | -0.076283 |

TABLE S22.
LINEAR RELATIONSHIPS BETWEEN PARAMETERS AND COORDINATE POINTS BY SCATTER PLOTS ANALYSIS.

**Form 2 (Second Middle).**

| | NL | SP | x1 | y1 | x2 | y2 | x3 | y3 |
|---|---|---|---|---|---|---|---|---|
| $R_0$ | 1% | m | -0.46396 | 4.3692 | 5.3913 | -9.4054 | -4.1106 | 1.8783 |
| | | b | 671.56 | 104.67 | -1087.6 | 1095.8 | 1566.6 | 459.3 |
| | | r | -0.071628 | 0.16004 | **0.76278** | -0.27625 | -0.49999 | 0.016513 |
| | 2% | m | -0.34015 | 8.5384 | 6.0897 | -8.2758 | -4.3077 | -19.42 |
| | | b | 630.63 | -269.41 | -1289.3 | 1028.2 | 1622.4 | 930.69 |
| | | r | -0.053085 | 0.3356 | **0.75643** | -0.21806 | -0.49183 | -0.19005 |
| | 5% | m | 0.10488 | 5.3276 | 6.9931 | -8.0269 | -6.2405 | 0.91103 |
| | | b | 486.5 | 42.068 | -1541.4 | 1035.4 | 2142.1 | 505.25 |
| | | r | 0.014079 | 0.17406 | **0.72258** | -0.17997 | -0.6076 | 0.0076089 |
| | 10% | m | 2.5548 | 36.899 | 17.389 | -23.858 | -7.5058 | 16.904 |
| | | b | -268.64 | -2642.4 | -4437.4 | 2207.1 | 2610.7 | 308.28 |
| | | r | 0.063673 | **0.23121** | **0.32942** | -0.093872 | -0.11668 | 0.023779 |
| $R_\infty$ | 1% | m | 0.086294 | -0.86733 | -0.41008 | 0.37255 | 1.4773 | -5.3463 |
| | | b | 218.15 | 328.54 | 370.77 | 226.4 | -133.19 | 367.01 |
| | | r | 0.067889 | -0.1619 | -0.29566 | 0.055762 | **0.9157** | -0.23951 |
| | 2% | m | 0.057691 | -0.79055 | -0.43163 | 0.53808 | 1.466 | 3.5253 |
| | | b | 228.44 | 321.43 | 376.91 | 215.71 | -130.47 | 172.48 |
| | | r | 0.049465 | -0.17071 | -0.29457 | 0.077893 | **0.91958** | 0.18955 |
| | 5% | m | -0.068522 | -0.25555 | -0.71473 | 0.9206 | 1.6192 | -0.60832 |
| | | b | 274.34 | 272.23 | 460.28 | 190.55 | -170.46 | 262.38 |
| | | r | -0.054652 | -0.049607 | -0.4388 | 0.12264 | **0.93669** | -0.030187 |
| | 10% | m | 0.22934 | 0.011517 | -0.89154 | 0.29867 | 1.5771 | 2.9987 |
| | | b | 157.69 | 241.7 | 505.04 | 223.6 | -163.21 | 177.02 |
| | | r | 0.13743 | 0.0017351 | **-0.40609** | 0.028255 | **0.58952** | 0.10143 |
| $\alpha$ | 1% | m | 0.001152 | -0.0056161 | -0.013544 | 0.022596 | 0.012646 | -0.016129 |
| | | b | 0.37563 | 1.3093 | 4.7898 | -0.62976 | -2.4794 | 1.1537 |
| | | r | 0.067217 | -0.077746 | **-0.72417** | 0.25083 | **0.58132** | -0.053589 |
| | 2% | m | 0.00029395 | -0.012937 | -0.013879 | 0.01976 | 0.012919 | 0.044771 |
| | | b | 0.68755 | 1.9696 | 4.886 | -0.45268 | -2.5544 | -0.18494 |
| | | r | 0.018859 | -0.20903 | **-0.70874** | 0.21403 | **0.60636** | 0.18012 |
| | 5% | m | -0.0010332 | 0.0014411 | -0.010531 | 0.012699 | 0.011074 | 0.0039163 |
| | | b | 1.1484 | 0.63646 | 3.8794 | -0.039937 | -2.102 | 0.68135 |
| | | r | -0.084538 | 0.028699 | **-0.6633** | 0.17355 | **0.65723** | 0.019938 |
| | 10% | m | 0.00040748 | -0.0015888 | -0.0072734 | -0.002539 | 0.0042985 | 0.017877 |
| | | b | 0.55422 | 0.84833 | 2.8453 | 0.86798 | -0.4011 | 0.31355 |
| | | r | 0.043914 | -0.043046 | **-0.59581** | -0.043198 | **0.28895** | 0.10874 |
| $\tau$ | 1% | m | -2.89E-08 | 2.20E-07 | 3.21E-07 | -6.13E-07 | -2.94E-07 | 7.54E-08 |

| | | | | | | | |
|---|---|---|---|---|---|---|---|
| | b | 1.64E-05 | -1.42E-05 | -8.89E-05 | 4.45E-05 | 8.20E-05 | 4.06E-06 |
| | r | -0.069892 | 0.12631 | **0.71191** | -0.28216 | **-0.56059** | 0.010393 |
| 2% | m | -2.54E-08 | 5.29E-07 | 3.87E-07 | -6.26E-07 | -3.39E-07 | -1.80E-06 |
| | b | 1.55E-05 | -4.18E-05 | -0.00010797 | 4.57E-05 | 9.40E-05 | 4.56E-05 |
| | r | -0.05526 | 0.29016 | **0.67175** | -0.23026 | **-0.53986** | -0.2458 |
| 5% | m | 4.84E-08 | 6.60E-07 | 6.58E-07 | -9.67E-07 | -7.00E-07 | 2.05E-07 |
| | b | -7.97E-06 | -5.00E-05 | -0.00018465 | 7.14E-05 | 0.00019115 | 5.38E-06 |
| | r | 0.054347 | 0.18043 | **0.56919** | -0.18152 | **-0.57006** | 0.014359 |
| 10% | m | 0.0030941 | 0.014857 | 0.0040448 | -0.017842 | -0.0044616 | -0.030553 |
| | b | -1.1039 | -1.2939 | -1.1467 | 1.1864 | 1.1918 | 0.71299 |
| | r | **0.16449** | **0.19858** | **0.16345** | **-0.14974** | **-0.14795** | -0.09168 |

TABLE S23.
LINEAR RELATIONSHIPS BETWEEN PARAMETERS AND COORDINATE POINTS BY SCATTER PLOTS ANALYSIS.

**Form 3 (First third).**

| | NL | SP | x1 | y1 | x2 | y2 | x3 | y3 |
|---|---|---|---|---|---|---|---|---|
| $R_0$ | 1% | m | 80.357 | -263.86 | -165.69 | -606.55 | 22.617 | 98.962 |
| | | b | -38501 | 7778.7 | 78811 | 30937 | -9265.9 | -6378.6 |
| | | r | **0.22357** | -0.041498 | **-0.41595** | **-0.17123** | 0.055636 | 0.042476 |
| | 2% | m | 577.29 | 7753.9 | -274.17 | -1936 | -479.75 | 2191.8 |
| | | b | -2.79E+05 | -2.05E+05 | 1.31E+05 | 98553 | 2.14E+05 | -1.55E+05 |
| | | r | **0.20983** | **0.16043** | -0.088368 | -0.0719 | **-0.14276** | 0.12314 |
| | 5% | m | -0.97947 | 14.059 | -7.9146 | -13.734 | 5.7667 | 20.112 |
| | | b | 1098.1 | 239.32 | 4381.4 | 1297.7 | -1924.5 | -837 |
| | | r | -0.038613 | 0.037324 | **-0.29982** | -0.060634 | **0.23723** | **0.15444** |
| | 10% | m | 0.56002 | -58.267 | 1.8851 | 4.2081 | 6.9141 | 5.5268 |
| | | b | 308.94 | 2126 | -323.77 | 378.87 | -2384.7 | 201.83 |
| | | r | 0.037776 | -0.22771 | 0.10485 | 0.036684 | **0.47908** | 0.061221 |
| $R_\infty$ | 1% | m | -2.4368 | 9.8344 | 3.4763 | 25.13 | -10.03 | 24.934 |
| | | b | 1467.8 | 15.451 | -1360.2 | -973.32 | 4715.3 | -1516.1 |
| | | r | -0.059185 | 0.013502 | 0.076184 | 0.061931 | **-0.21538** | 0.093427 |
| | 2% | m | -0.051234 | -74.058 | -2.4971 | 39.525 | -2.3924 | -16.036 |
| | | b | 345.78 | 2297.4 | 1497.4 | -1645.4 | 1378.3 | 1474.6 |
| | | r | -0.0021878 | -0.18002 | -0.094559 | **0.17246** | -0.083637 | **-0.10585** |
| | 5% | m | -1.4914 | -11.046 | 0.38582 | -5.0353 | 3.2993 | 2.4223 |
| | | b | 1090.2 | 652.5 | 172.87 | 606.32 | -1097.2 | 181.4 |
| | | r | **-0.11825** | -0.058985 | 0.029396 | -0.044713 | **0.27299** | 0.037413 |
| | 10% | m | -1.1973 | -0.22958 | 1.2362 | -1.1973 | 1.7963 | -2.3899 |
| | | b | 939.43 | 338.55 | -268.57 | 393.38 | -441.06 | 501.58 |
| | | r | **-0.3062** | -0.0034014 | **0.26066** | -0.039568 | **0.47186** | -0.10036 |
| $\alpha$ | 1% | m | -0.023389 | 0.035698 | 0.039471 | 0.082746 | -0.017844 | -0.054855 |
| | | b | 12.213 | -0.16174 | -17.807 | -3.329 | 8.6855 | 4.7369 |

| | | | | | | | | |
|---|---|---|---|---|---|---|---|---|
| | | r | -0.47208 | 0.04073 | **0.71883** | 0.16946 | -0.31844 | -0.17081 |
| | 2% | m | -0.0096889 | 0.04327 | 0.017379 | 0.065523 | -0.0083635 | 0.03364 |
| | | b | 5.4425 | -0.43288 | -7.4672 | -2.5377 | 4.4186 | -1.6984 |
| | | r | **-0.26081** | 0.066301 | **0.41485** | **0.18022** | **-0.18431** | 0.13997 |
| | 5% | m | -0.0012712 | 0.046142 | 0.0047651 | 0.011711 | -0.0028854 | 0.0057024 |
| | | b | 1.2333 | -0.6289 | -1.659 | 0.026672 | 1.8791 | 0.19588 |
| | | r | -0.058728 | **0.14357** | **0.21155** | 0.060596 | **-0.13911** | 0.05132 |
| | 10% | m | -0.00047065 | -0.036477 | 0.0018804 | -0.0018188 | 0.0017779 | 0.0038319 |
| | | b | 0.94906 | 1.6703 | -0.2038 | 0.80296 | -0.055167 | 0.43942 |
| | | r | -0.05626 | **-0.25261** | **0.18533** | -0.028096 | **0.21831** | 0.075217 |
| $\tau$ | 1% | m | -0.0018381 | 0.023639 | 0.0036642 | 0.020955 | 0.00017857 | -0.00078126 |
| | | b | 0.89235 | -0.6358 | -1.7318 | -1.0485 | -0.084287 | 0.050928 |
| | | r | **-0.15651** | **0.11378** | **0.28151** | **0.18104** | 0.013443 | -0.010262 |
| | 2% | m | -0.70879 | -9.7354 | 0.31439 | 2.5265 | 0.60361 | -2.7151 |
| | | b | 343.25 | 257.74 | -150.22 | -127.77 | -268.88 | 193.26 |
| | | r | **-0.20701** | **-0.16185** | 0.081425 | 0.075397 | **0.14433** | **-0.12257** |
| | 5% | m | -3.78E-07 | 0.00020303 | -1.14E-05 | 0.00010651 | -6.12E-06 | 5.80E-06 |
| | | b | 0.00026263 | -0.0053657 | 0.0054952 | -0.0052099 | 0.0027711 | -0.00034266 |
| | | r | -0.0047771 | **0.17258** | **-0.13815** | **0.15056** | -0.080558 | 0.01426 |
| | 10% | m | -2.44E-07 | -2.65E-05 | 5.53E-07 | 1.78E-06 | 3.12E-06 | 1.98E-06 |
| | | b | 0.0001669 | 0.00074009 | -0.0002255 | -4.70E-05 | -0.0012987 | -9.70E-05 |
| | | r | -0.035217 | **-0.22158** | 0.06584 | 0.033167 | **0.46234** | 0.047053 |

TABLE S24.
LINEAR RELATIONSHIPS BETWEEN PARAMETERS AND COORDINATE POINTS BY SCATTER PLOTS ANALYSIS.

| Form 3 (middle third). | | | | | | | | |
|---|---|---|---|---|---|---|---|---|
| | **NL** | **SP** | **x1** | **y1** | **x2** | **y2** | **x3** | **y3** |
| $R_0$ | 1% | m | 5.6954 | 15.165 | -2.9802 | -16.929 | -0.18798 | 3.8162 |
| | | b | -1891.7 | -759.09 | 1687.8 | 2000.1 | 561.38 | 179.47 |
| | | r | **0.78119** | 0.38774 | -0.37244 | -0.45076 | -0.021719 | 0.10228 |
| | 2% | m | 5.0983 | 12.349 | -2.6002 | -7.1793 | -1.4022 | 4.5326 |
| | | b | -1641.3 | -522.11 | 1539 | 1138.2 | 967.58 | 122.57 |
| | | r | **0.68862** | 0.31819 | -0.34989 | -0.19787 | -0.14582 | 0.12399 |
| | 5% | m | 2.8852 | 7.9041 | -0.064108 | -16.149 | -0.17596 | -0.096817 |
| | | b | -704.65 | -141.93 | 537.49 | 1946.1 | 570.55 | 520.11 |
| | | r | **0.46001** | **0.22429** | -0.010065 | **-0.57982** | -0.024155 | -0.0034673 |
| | 10% | m | 1.8234 | 5.2282 | 0.39985 | -5.2609 | -0.7363 | 2.5671 |
| | | b | -250.33 | 115.15 | 386.2 | 1011.3 | 790.52 | 331.46 |
| | | r | **0.48579** | **0.297** | 0.10962 | -0.27459 | -0.1696 | 0.14266 |
| $R_\infty$ | 1% | m | -4.7606 | -12.437 | 2.8279 | 16.754 | 1.1313 | -5.7218 |
| | | b | 2249.2 | 1282.7 | -877.23 | -1234.8 | -124.86 | 729.91 |
| | | r | **-0.71747** | -0.3494 | 0.38832 | 0.49017 | 0.14361 | -0.16851 |

| | | | | | | | | |
|---|---|---|---|---|---|---|---|---|
| | 2% | m | -4.1639 | -9.5352 | 2.593 | 9.2124 | 2.3068 | -7.7056 |
| | | b | 1998.5 | 1038.7 | -786.18 | -568.31 | -517.78 | 893.22 |
| | | r | **-0.59946** | -0.26187 | 0.37192 | 0.27063 | 0.2557 | -0.22468 |
| | 5% | m | -1.8118 | -1.9151 | 0.52159 | 17.101 | 1.1501 | -5.1926 |
| | | b | 1003.4 | 397.85 | 32.241 | -1279.3 | -143.09 | 672.86 |
| | | r | -0.29629 | -0.055742 | 0.083993 | **0.6298** | 0.16193 | -0.19074 |
| | 10% | m | -0.69449 | -2.0921 | -0.086088 | 7.7628 | 2.138 | -5.3465 |
| | | b | 525.31 | 394.38 | 256.76 | -466.81 | -493.24 | 665.31 |
| | | r | -0.17253 | -0.11082 | -0.022007 | **0.37782** | **0.4592** | -0.27706 |
| $\alpha$ | 1% | m | -0.025975 | -0.0636 | 0.01391 | 0.080645 | 0.0011393 | -0.027388 |
| | | b | 11.714 | 6.0867 | -4.7385 | -6.3408 | 0.43213 | 3.1034 |
| | | r | **-0.75549** | -0.34483 | 0.36862 | 0.45535 | 0.027914 | -0.15566 |
| | 2% | m | -0.006613 | -0.022911 | -0.00091095 | 0.050917 | 0.0088806 | 0.0041152 |
| | | b | 3.5394 | 2.6594 | 1.1223 | -3.7516 | -2.1883 | 0.41434 |
| | | r | -0.22768 | -0.15048 | -0.031247 | **0.35773** | 0.23541 | 0.028696 |
| | 5% | m | -0.0014134 | -0.0072693 | -0.0019821 | 0.025587 | -0.0021447 | -0.010645 |
| | | b | 1.2624 | 1.2678 | 1.4536 | -1.6059 | 1.3796 | 1.555 |
| | | r | -0.086875 | -0.079523 | -0.11997 | **0.35416** | -0.1135 | -0.14696 |
| | 10% | m | -0.0017833 | -0.0041673 | -0.001838 | 0.025909 | 0.0027379 | -0.0094234 |
| | | b | 1.437 | 1.002 | 1.3853 | -1.6415 | -0.25675 | 1.4402 |
| | | r | -0.18942 | -0.09438 | -0.20089 | **0.53915** | 0.25143 | -0.20878 |
| $\tau$ | 1% | m | 6.86E-08 | 2.08E-07 | -5.09E-08 | -9.39E-08 | -1.85E-08 | -1.19E-07 |
| | | b | -2.32E-05 | -1.16E-05 | 2.59E-05 | 1.39E-05 | 1.18E-05 | 1.55E-05 |
| | | r | **0.72903** | 0.41147 | -0.49286 | -0.19373 | -0.16564 | -0.24629 |
| | 2% | m | 1.63E-07 | 3.04E-07 | -1.30E-07 | 1.60E-07 | 6.11E-09 | 6.48E-08 |
| | | b | -6.30E-05 | -1.98E-05 | 5.72E-05 | -8.78E-06 | 3.36E-06 | -4.34E-08 |
| | | r | **0.4798** | 0.17101 | -0.38207 | 0.096236 | 0.013873 | 0.038731 |
| | 5% | m | 1.02E-07 | 4.27E-07 | -5.35E-08 | -2.34E-07 | -1.77E-08 | -3.76E-07 |
| | | b | -3.83E-05 | -3.04E-05 | 2.62E-05 | 2.57E-05 | 1.08E-05 | 3.63E-05 |
| | | r | **0.38841** | 0.28838 | -0.19991 | -0.20004 | -0.057748 | -0.32077 |
| | 10% | m | 5.88E-08 | 2.26E-07 | -2.45E-08 | 9.53E-08 | -2.54E-08 | -1.71E-07 |
| | | b | -1.93E-05 | -1.23E-05 | 1.59E-05 | -2.18E-06 | 1.48E-05 | 2.04E-05 |
| | | r | **0.40909** | 0.33586 | -0.1751 | 0.12994 | -0.15281 | -0.24781 |

TABLE S25.
LINEAR RELATIONSHIPS BETWEEN PARAMETERS AND COORDINATE POINTS BY SCATTER PLOTS ANALYSIS.

**Form 3 (last third).**

| | NL | SP | x1 | y1 | x2 | y2 | x3 | y3 |
|---|---|---|---|---|---|---|---|---|
| $R_0$ | 1% | m | -85.996 | 422.86 | 28.236 | 14.766 | -16.735 | -150.12 |
| | | b | 23987 | -17225 | -6786.7 | 206.95 | 4801.4 | 2513.1 |
| | | r | **-0.13723** | **0.10182** | 0.043046 | 0.0016916 | -0.023784 | -0.011194 |
| | 2% | m | -14.734 | -123.93 | -16.783 | 287.77 | -23.983 | -311.17 |

| | | | | | | | | |
|---|---|---|---|---|---|---|---|---|
| | | **b** | 4453.9 | 5633.7 | 4788.1 | -5885.8 | 6565.8 | 4540.8 |
| | | **r** | **-0.11777** | **-0.15065** | **-0.10185** | **0.19779** | **-0.15743** | **-0.1237** |
| | **5%** | **m** | -1.3051 | -16.815 | -3.8009 | -6.4115 | -6.255 | -30.418 |
| | | **b** | 723.03 | 1070.4 | 1340.7 | 500.57 | 1948.2 | 761.96 |
| | | **r** | -0.055197 | **-0.12495** | **-0.16518** | -0.02425 | **-0.25509** | -0.072811 |
| | **10%** | **m** | 475.62 | 633.26 | 205.73 | -2892.2 | -381.8 | -4372.9 |
| | | **b** | -1.30E+05 | -20527 | -46127 | 67294 | 97187 | 62823 |
| | | **r** | **0.41827** | 0.10914 | 0.095708 | **-0.27943** | **-0.2417** | **-0.19776** |
| $R_\infty$ | **1%** | **m** | 6.4741 | 10.991 | -20.132 | 68.254 | 14.931 | -115.24 |
| | | **b** | -1537.6 | -233.09 | 5445.2 | -1266.1 | -3582.2 | 1750.5 |
| | | **r** | **0.20134** | 0.051574 | **-0.59811** | **0.15239** | **0.41353** | **-0.16746** |
| | **2%** | **m** | 2.2707 | 26.808 | -11.481 | 30.653 | 5.3497 | -14.987 |
| | | **b** | -398.04 | -903.57 | 3203.6 | -450.57 | -1146.6 | 420.14 |
| | | **r** | 0.11435 | **0.20531** | **-0.43898** | **0.13273** | **0.22125** | -0.037535 |
| | **5%** | **m** | 0.13073 | -8.1363 | -2.3275 | 12.77 | -1.4898 | -12.241 |
| | | **b** | 200.63 | 580.45 | 837.17 | -42.048 | 615.1 | 398.5 |
| | | **r** | 0.023566 | **-0.25768** | **-0.43112** | **0.20587** | **-0.25896** | **-0.12489** |
| | **10%** | **m** | 0.22441 | 2.833 | -2.0546 | 18.613 | -0.79531 | 18.457 |
| | | **b** | 150.06 | 98.581 | 726.94 | -185.45 | 405.4 | -29.142 |
| | | **r** | 0.070285 | **0.17388** | **-0.3404** | **0.64043** | **-0.1793** | **0.29727** |
| $\alpha$ | **1%** | **m** | 0.01024 | 0.051434 | -0.086027 | 0.33406 | 0.061911 | -0.70156 |
| | | **b** | -2.0618 | -1.4283 | 23.024 | -6.5829 | -15.07 | 9.9976 |
| | | **r** | 0.074991 | 0.056836 | **-0.60186** | 0.17563 | 0.4038 | -0.24006 |
| | **2%** | **m** | 0.00058261 | 0.11746 | -0.037 | 0.26334 | 0.022952 | 0.025319 |
| | | **b** | 0.54032 | -4.2327 | 10.308 | -5.0798 | -5.1727 | 0.36495 |
| | | **r** | 0.0079152 | **0.24268** | **-0.38167** | **0.30764** | **0.25608** | 0.017107 |
| | **5%** | **m** | 0.013589 | -0.043694 | -0.016662 | 0.012037 | -0.010805 | 0.023725 |
| | | **b** | -3.0803 | 2.5391 | 4.9911 | 0.43136 | 3.4369 | 0.38121 |
| | | **r** | **0.33741** | **-0.19062** | **-0.42512** | 0.026731 | **-0.2587** | 0.033342 |
| | **10%** | **m** | -0.0096478 | 0.041121 | -0.0035084 | 0.091995 | 0.0096397 | -0.034676 |
| | | **b** | 3.303 | -1.1143 | 1.4353 | -1.4144 | -1.7621 | 1.0158 |
| | | **r** | **-0.58762** | **0.49081** | -0.11304 | **0.61557** | **0.42265** | -0.10861 |
| $\tau$ | **1%** | **m** | -1.89E-05 | 8.31E-05 | 4.05E-06 | 1.88E-05 | -7.91E-06 | -4.88E-05 |
| | | **b** | 0.0051862 | -0.0034599 | -0.0010201 | -0.00038118 | 0.0020498 | 0.00067549 |
| | | **r** | **-0.15259** | **0.10126** | 0.03127 | 0.010899 | -0.056884 | -0.018419 |
| | **2%** | **m** | 2.63E-07 | -3.14E-05 | -2.45E-06 | 1.27E-05 | -6.76E-06 | -2.46E-05 |
| | | **b** | -5.80E-05 | 0.0013313 | 0.00064881 | -0.00026493 | 0.0017437 | 0.00033929 |
| | | **r** | 0.013765 | **-0.25004** | -0.097293 | 0.057209 | **-0.29096** | -0.064215 |
| | **5%** | **m** | -3.11E-07 | -2.85E-07 | -3.32E-07 | -1.97E-06 | -6.96E-07 | -4.33E-06 |
| | | **b** | 9.13E-05 | 1.70E-05 | 9.06E-05 | 4.81E-05 | 0.00018171 | 6.21E-05 |
| | | **r** | **-0.12948** | -0.02082 | **-0.14218** | -0.07354 | **-0.27968** | -0.10213 |

| | | | | | | | |
|---|---|---|---|---|---|---|---|
| | **m** | 1169.1 | 1688.5 | 524.35 | -7072.9 | -920.93 | -11225 |
| **10%** | **b** | -3.21E+05 | -56887 | -1.19E+05 | 1.64E+05 | 2.34E+05 | 1.60E+05 |
| | **r** | **0.41067** | 0.11624 | 0.09744 | **-0.27296** | **-0.23288** | **-0.20277** |

## VII. SUPPORTING INFORMATION REFERENCE


[1] S. Grimnes and O. Martinsen, "Data and Models," in *Bioimpedance and Bielectricity Basics*, Second., Elsevier, 2008, pp. 283–332.

[2] N. Reljin *et al.*, "Machine Learning Model Based on Transthoracic Bioimpedance and Heart Rate Variability for Lung Fluid Accumulation Detection: Prospective Clinical Study.," *JMIR Med. informatics*, vol. 8, no. 8, p. e18715, Aug. 2020, doi: 10.2196/18715.

[3] C. A. González-Correa, E. Colina-Gallo, and D. A. Miranda-Mercado, "The alpha parameter of the Cole-Cole model as an indicator of fibromyalgia," *J. Phys. Conf. Ser.*, vol. 1272, no. 1, p. 012003, Jul. 2019, doi: 10.1088/1742-6596/1272/1/012003.

[4] S. Kun, B. Ristic, R. A. Peura, and R. M. Dunn, "Real-time extraction of tissue impedance model parameters for electrical impedance spectrometer," *Med. Biol. Eng. Comput.*, vol. 37, no. 4, pp. 428–432, Jul. 1999, doi: 10.1007/BF02513325.

[5] D. Ayllon, F. Seoane, and R. Gil-Pita, "Cole equation and parameter estimation from electrical bioimpedance spectroscopy measurements - A comparative study," 2009, doi: 10.1109/IEMBS.2009.5334494.
